# Correlation between patient-specific quality assurance in volumetric modulated arc therapy and 2D dose image features

**DOI:** 10.1038/s41598-023-30719-4

**Published:** 2023-03-10

**Authors:** Yixiao Guo, Jinyan Hu, Yang Li, Juntao Ran, Hongyi Cai

**Affiliations:** 1 Department of Radiation Oncology, Gansu Provincial Hospital, Lanzhou, 730000 People’s Republic of China; 2 Department of Oncology, Longhua District Peopleʼs Hospital, Shenzhen, 518109 People’s Republic of China; 3 Department of Radiation Oncology, Weifang Peopleʼs Hospital, Weifang, 261000 People’s Republic of China; 4 Department of Radiation Oncology, The First Hospital of Lanzhou University, Lanzhou, 730000 People’s Republic of China

**Keywords:** Biomedical engineering, Optical physics, Cancer

## Abstract

In radiotherapy, air-filled ion chamber detectors are ubiquitously used in routine dose measurements for treatment planning. However, its use has been restricted by intrinsic low spatial resolution barriers. We developed one procedure for patient-specific quality assurance (QA) in arc radiotherapy by coalescing two adjacent measurement images into a single image to improve spatial resolution and sampling frequency, and investigated how different spatial resolutions affect the QA results. PTW 729 and 1500 ion chamber detectors were used for dosimetric verification via coalescing two measurements with 5 mm-couch shift and the isocenter, and only isocenter measurement, which we call coalescence and standard acquisition (SA). Statistical process control (SPC), process capability analysis (PCA), and receiver operating characteristic (ROC) curve were used to compare the performance of the two procedures in determining tolerance levels and identifying clinically relevant errors. By analyzing 1256 γ values calculated on interpolated data points, our results indicated that detector 1500 showed higher averages in coalescence cohorts at different tolerance criteria and the dispersion degrees were spread out smaller. Detector 729 yielded a slightly lower process capability of 0.79, 0.76, 1.10, and 1.34, but detector 1500 exhibited somewhat different results of 0.94, 1.42, 1.19, and 1.60 in magnitude. The results of SPC individual control chart showed that cases in coalescence cohorts with γ values lowering its lower control limit (LCL) were greater than those in SA cohorts for detector 1500. A combination of the width of multi-leaf collimator (MLC) leaf, the cross-sectional area of the single detector, and the spacing between adjacent detectors might lead to discrepancies in percent γ values across diverse spatial resolution scenarios. The accuracy of reconstructed volume dose is mainly determined by the interpolation algorithm used in dosimetric systems. The magnitude of filling factor in the ion chamber detectors determined its ability to detect dose deviations. SPC and PCA results indicated that coalescence procedure could detect more potential failure QA results than SA while enhancing action thresholds.

## Introduction

As a challenging and thorough QA area in radiotherapy, radiotherapy dosimetry and plan verification is a sophisticated process undertaken by medical physicist^1^. These checks are primarily intended to identify obvious errors and ensure the appropriateness of treatment^2,3^. As such, it is a vital component of radiation oncology safety and an area with potential for machine learning-based tools to achieve high clinical impact^1^. 2D ion chamber detectors are widely used in radiotherapy dosimetry because of superior dosimetric characteristics and convenience of data readout, however, to date they suffer from inferior spatial resolution. The high cost limits the number, size, and arrangement of detectors^4–6^. An insufficient spatial resolution may affect the slope of the measured profiles in wider photon fields, it also affects a narrower photon fieldʼs center dose. This can lead to limited sampling of the radiation beam in regions of high dose gradients, and under-sampling negatively influences the measurement precision and, subsequently, the accuracy of the reconstructed volumetric dose. To address this limitation, the resolution of ion chamber detectors (PTW Freiburg, Germany) could be meliorated by coalescing two or multiple acquisition sequences as well as increasing the spatial sampling frequency and the coverage of dose distribution with the sensitive areas of ionization chambers^7,8^. Using the coalescence, prior studies have discovered evidence for improvement in the QA procedures in both intensity modulated radiation therapy (IMRT) and stereotactic body radiotherapy (SBRT) with 10MV flatting filter free (FFF) energy^4,5,7,9–11^. Due to drastic variations in the modulated delivery techniques between volumetric modulated arc therapy (VMAT) and IMRT^12^, the penumbra perturbation and penetration power between 6 MV low-energy and 10 MV mid-energy photons are vastly different^13^. It is still unclear whether the coalescing procedure can enhance the QA procedure of 6MV arc deliveries.

Herein, we used ion chamber detectors (PTW Freiburg, Germany) to perform dosimetric measurements for 6MV VMAT plans. By spatially and temporally overlapping two dose-maps, whether increased spatial sampling could contribute to the improvement of patient-specific QA process was evaluated using statistical process control and process capability analysis. Eliminate the impact of calculation type, we aimed to investigate how different spatial resolutions and sampling frequency affect QA results. Across diverse spatial resolution scenarios, the sensitivities in identifying clinically relevant systematic and random errors, as well as the dose profiles at different off-axis positions, were evaluated.

## Methods

### Case selection, treatment plan preparation, and delivery

VMAT plans were retrospectively selected from various anatomical sites of 126 patients (37 head/neck, 44 mediastinum/lungs, 47 abdomen/pelvis) who had completed treatment courses from our institution database. The prescription doses are 50–73.92 Gy/25–33 fraction for head/neck plans, 50–66 Gy/25–33 fraction for mediastinum/lung plans, and 45–62.72 Gy/25–28 fraction for abdomen/pelvis plans, respectively. The field sizes range from 8.6 to 14.9 cm in X direction and 11.3 to 21 cm in Y direction for head/neck plans, 4.9 to 14.9 cm in X direction and 9.8 to 21 cm in Y direction for mediastinum/lung plans, and 11.8 to 14.9 cm in X direction and 13.6 to 21 cm in Y direction for abdomen/pelvis plans, respectively. Following the guidelines of AAPM Task Group 119^14,15^, VMAT test plans have included mutiple-targets and C shape planning target volumes (PTVs). Eclipse treatment planning system (TPS, version 13.6) was used for all delineation on PTVs and organs at risk (OARs), and dose calculations were performed with a 6 MV photon beam. The high definition (HD) 120 muti-leaf collimator, integrated as a tertiary collimator in EDGE linac (Varian Medical System, Palo Alto, CA), consisted of 60 tungsten leaf pairs, of which the innermost 32 leaf pairs were 2.5 mm wide, and the outer 28 leaf pairs were 5.0 mm wide. Optimization used the photon optimizer (PO, version 13.6.23), while dose calculation utilized an anisotropic analytical algorithm (AAA, version 13.6.23) with a grid resolution of 2.5 × 2.5 × 2.5 mm^3^.

### Ethics approval and consent to participate

All experimental protocols were approved by the Ethics Committee of the Gansu Provincial Hospital, and the Registration number is ChiCTR2100054530. The Ethics Committee of the Gansu Provincial Hospital exempt from the informed consent requirement, for the following reasons: (1) All procedures performed in this study involve radiotherapy planning based on human computed tomography (CT) images without human participants; (2) This study involved retrospective data collection, and we know that many patients have died since we collected the data, so informed consent cannot be obtained; (3) patient data were fully anonymized and they were nonidentifiable.

### Methods statement

All methods were carried out in accordance with relevant guidelines and regulations.

### Ethical statement

The procedures used in studies were in accordance with the ethical standards of the institutional and/or national research committees and with the Helsinki Declaration of 1964, and its later amendments or comparable ethical standards.

### Ion chamber detectors

Detectors 729 and 1500 (PTW Freiburg, Germany, Octavius specifications) were used in current study. The detector 729 had 729 vented ion chambers uniformly arranged over an area of 27 × 27 cm^2^, each with a cross-sectional area of 5 × 5 mm^2^ and an air-filled volume height of 5 mm^16^. An edge-to-edge distance of 5 mm separated the ion chambers equidistantly^13^. The detector 1500 consisted of 1405 vented ion chambers, each with an active area of 4.4 × 4.4 mm^2^, a height of 3 mm, and a center-to-center distance of 7.07 mm^7^. This detector generated spatial sampling frequencies of 0.1 mm^−1^ in each row or column, as well as 0.14 mm^−1^ diagonal, which doubled to 0.2 mm^–1^ along each row or column by coalescing two adjacent measurements^7^. Dose-maps between measured and calculated frequencies were evaluated using Verisoft software (version 7.1).

### The spatial sampling of ion chamber detector

**Figure 1:**
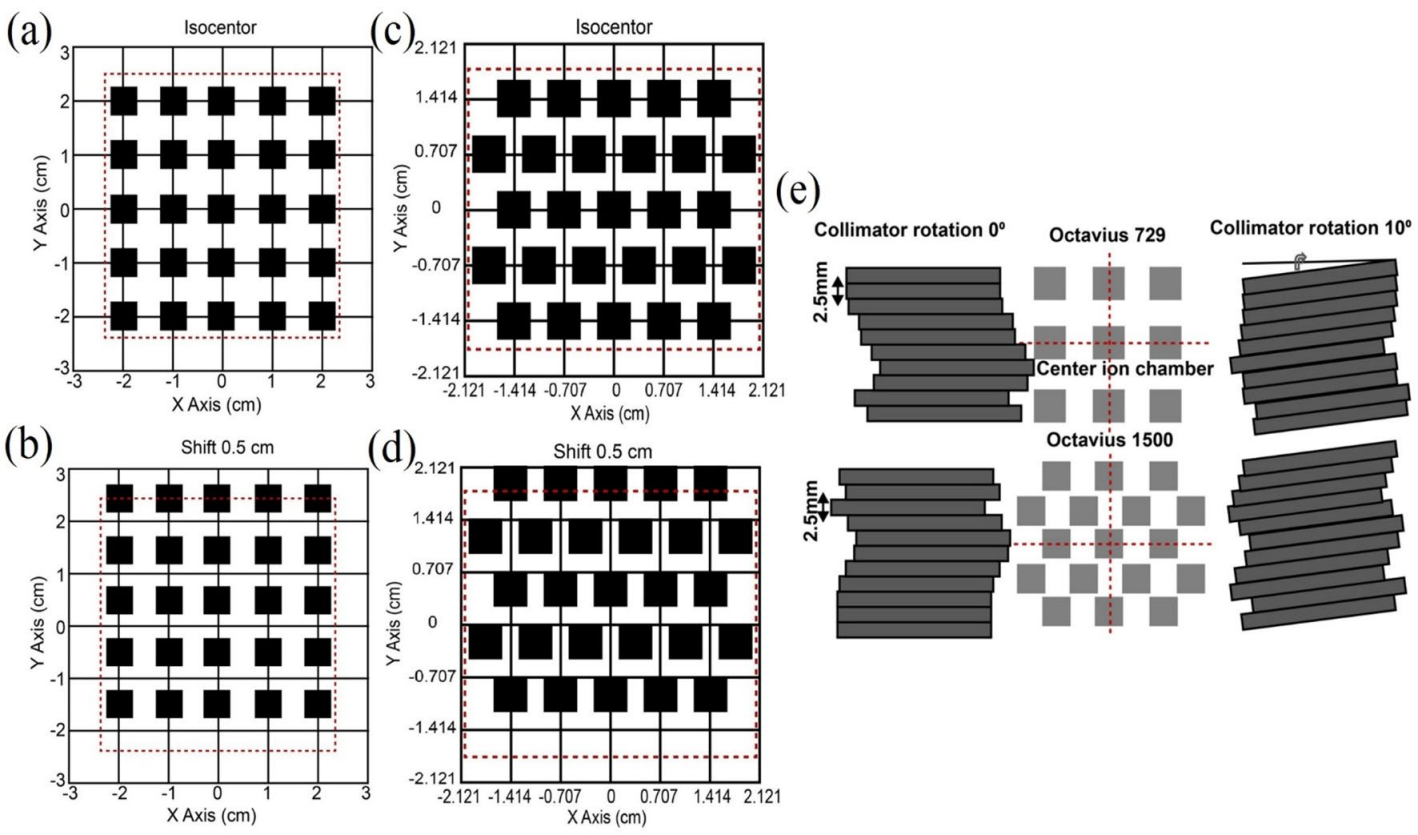
Schematic diagrams of sampling sequence for detectors 729 and 1500. **(a,c)** Were obtained by positioning detectors in the isocenter, i.e., standard acquisition. **(b,d)** Were obtained by longitudinal moving treatment couch + 0.5 cm. The square shape represents one single ion chamber. **(e)** Sampling schematic of ion chambers on inner 32 leaf pairs at multi-leaf collimator angle 0° (as typically set in IMRT) and 10° (as typically set in arc delivery). The dashed line indicates the central axis of beam across the center area with a row of detectors lateral and longitudinal, respectively. The position of the central ion chamber coincides with the isocenter. At collimator angle 0°, the lateral dashed line is aligned with the gap between a central pair of leafs.

As showed in Fig. 1a,c, during sampling, the first measurement was performed at the isocenter position (SA). The detector was subsequently shifted + 0.5 cm longitudinally (Fig. 1b,d). By coalescing both measurements, the total number of measurement points was increased from 729 to 1458, and the longitudinal spacing between neighboring detectors in detector 729 eliminated. Detector 1500 had more complex sampling where almost all detector spaces were eliminated by coalescence. Comparisons were performed between SA and coalescence in the longitudinal direction. In normal procedures, the crosshairs of the radiation field align with the marks outside the detector. The central axis intersects the central ion chamber, as shown in Fig. 1e. For inner 32 leaf pairs, a leaf pairs will directly overlap with a row of detectors if collimator at 0° (i.e., leaf motion parallel to gantry motion) during one standard measurement for detector 729. Then, readings of the ion chamber rows are associated with the leaf pairs, whereas abutting pair align with the gap between two rows of detectors and completely misses. When the measurement was acquired by longitudinally moving the linac couch + 5 mm, the completely missed leafs were sampled. All leafs were completely sampled after both measurements had subsequently coalesced. In an individual detector, leafs are oriented along with the gantry motion, and the entire penumbra and tail of two leaf tips are exposed to the dose regardless of whether they are moving. When the collimator rotates at 10°, a portion of each leaf is sampled during each measurement, but the total number of acquired leafs is the same as those of 0°, so it is possible for either the side or the tip of leafs to partially sweep a detector due to leaf movements that are not parallel with gantry motion. For detector 1500, a checkerboard pattern of arrangement and single ion chamber size allows sampling on most leafs at any collimator angle, and either side or tip of leafs completely sweep detectors by coalescence. The outer 28 leaf pairs are similar to above.

### Using the planning computed tomography as patient anatomy model for dose reconstruction

**Figure 2:**
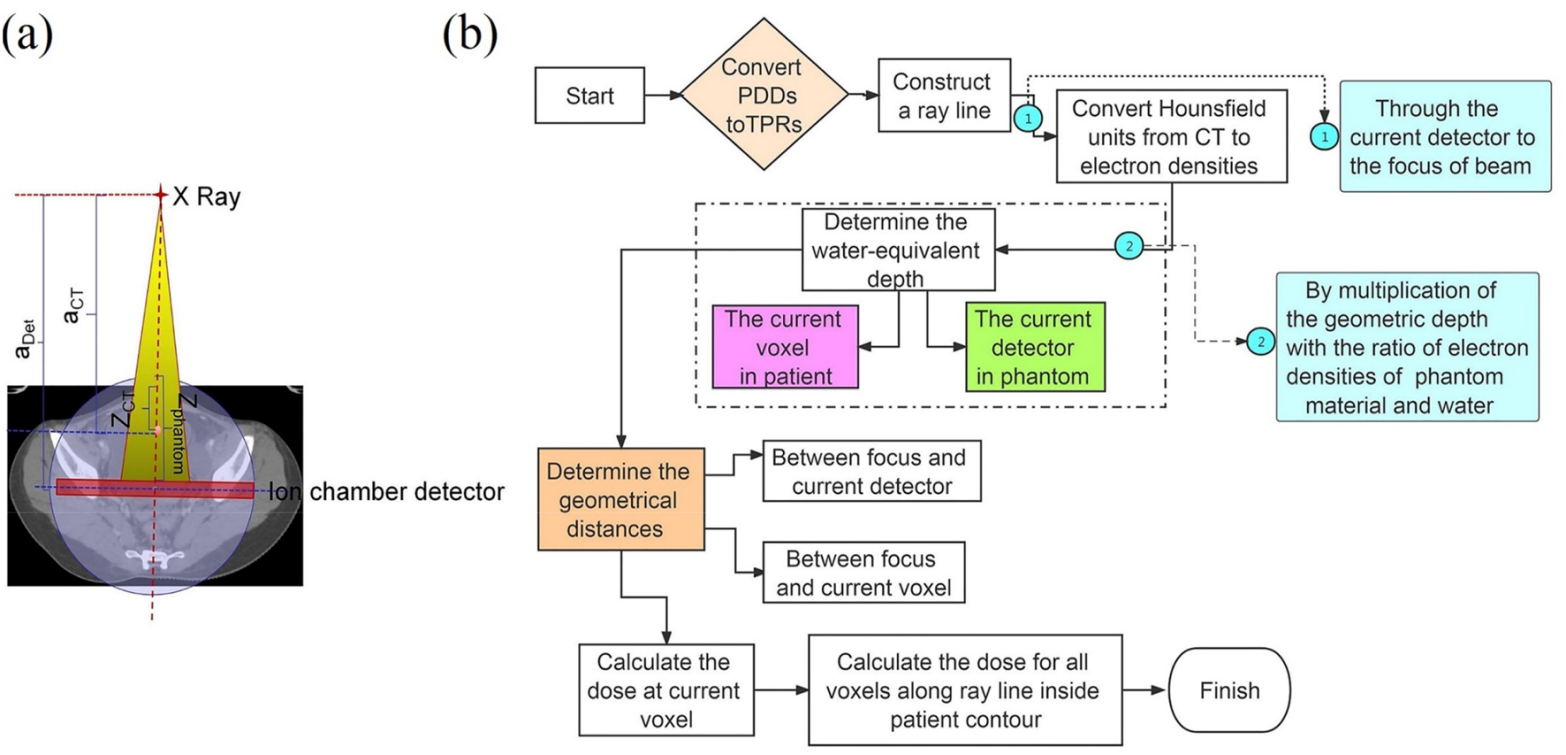
Reconstructing 3D dose on CT dataset. **(a)** Schematic illustration of dose reconstructed on an abdominal CT image. Dose measured by the current detector at the water-equivalent depth Z_Phantom_ corresponds to the dose of the current voxel at the water-equivalent depth Z_CT_ in CT image. a_Det_ and a_CT_ are geometrical distances from the focus of X-ray to the current detector and voxel, respectively. **(b)** Flow chart of the reconstruction dose.

After importing plan data containing Digital Imaging and Communications in Medicine (DICOM) radiotherapy (RT) plan, DICOM RT structures, and DICOM RT dose into Verisoft software, a 3D dose deposition on CT dataset was subsequently reconstructed using the measured dose-maps^5^. Human body is heterogeneous along the ray line, and the source-to-surface-distance differs because of irregular contour shape, as shown in Fig. 2a. The irradiation beam passes through an ion chamber detector embedded in a motorized cylindrical polystyrene phantom, corresponding to CT images (for example, the abdominal CT image). A relationship between the dose measured by the current detector, D_Det_, and the dose at a point along the ray line in CT image, D_CT_, is established to estimate the dose in the patient geometry. Figure 2b contains a flow chart of this algorithm.

The dose on CT image along a ray line through the current detector and the focus was reconstructed, which was given by^17,18^

1 $${D}_{CT}={D}_{Det}\cdot \frac{TPR\left({Z}_{CT}\right)}{TPR\left({Z}_{Det}\right)}\cdot {\left(\frac{{a}_{Det}}{{a}_{CT}}\right)}^{2}.$$

The first term accounts for different thicknesses of overlaying material ahead the current detector and the current voxel, and the second term accounts for different distances to the focus by applying the inverse square law. TPRs are tissue-phantom ratios transformed from percentage depth dose (PDD).

Water-equivalent depth ${Z}_{CT}$ was obtained from geometrical depth ${Z}_{CT}^{geom}$ and electron densities of the materials involved, as shown

2 $${Z}_{CT}={Z}_{CT}^{geom}\cdot \frac{\sum_{i=1}^{n}{\rho }_{CT,i}}{n\cdot {\rho }_{Water}},$$

in which n is the voxel number along ray line from the current voxel to the patient’s contour shape, ${\uprho }_{\mathrm{water}}$ is electron density of water, and ${\rho }_{CT, i}$ is electron density of voxel i (i = 1, 2, …, n).

### γ analysis

γ comparison method is accordance with the protocol of TG-218^21^, it is common to limit the γ calculation to points above a set threshold (TH) level, which could be 10% or 20% of the maximum dose value^19–21^. In this study, 10% of the maximum dose within the calculated volume was set according to manufacturer’s suggestion. All cases were evaluated using 3D γ metrics at 3%/2 mm, 2%/2 mm, 2%/1 mm, and 1%/1 mm tolerance criteria with global normalization.

### Statistical analysis

#### Statistical process control (SPC) and process capability analysis (PCA)

The SPC technique has been widely utilized to monitor the QA process of treatment planning, by which process-based tolerances and action limits were set according to the protocol of TG-218^21^. It also determines the effects of changes in methods or conditions during the course of a radiotherapy experiment, such as dosimetric changes to a treatment plan when using alternative techniques^22–24^. In general, statistical quality control consists of comparing a process’s current performance with its historical performance^25^. As a result of this study using SPC charts and PCA method, a statistical expectation was provided of how the coalescence cohort will perform in comparison to the SA cohort.

To monitor and control variations in the process, the individuals (*x*) and moving range (mR) control charts were used to indicate uncertainties in the data distribution. The percent γ value was considered as a quality characteristic of the QA process encompassing upper control limit (UCL), lower control limit (LCL), and center line (CL)^26,27^. Control limits, also known as tolerance limits, are warning indicators of potential changes in one process^28^. Introducing a new cause of variation may lead to observations falling outside the control limits, known as systematic error or special cause^24^. The center lines were defined as the average of a data group. As the maximum percent γ value is 100%, there is no clinical significance of a UCL when examining γ value. Therefore, LCL was the only parameter that defined the passing criteria for the percent γ value, i.e., tolerance limit^24^. Accordingly, one chart displays the individual measured values (*x* chart), while the other displays the difference between adjacent measurements (mR chart). LCL and CL for a given set of data were determined as follows^27,28^.

3 $$\overline{mR }=\frac{\sum_{i=2}^{n}\left|{x}_{\mathrm{i}}-{x}_{\mathrm{i}-1}\right|}{n-1},$$

4 $$LCL=\overline{x }-2.66\cdot \overline{mR },$$

5 $$CL=\overline{x }=\frac{\sum_{i=1}^{n}{x}_{i}}{\mathrm{n}}.$$

In which *x* represents individual γ value. $\overline{ mR }$ is the average moving range, i.e. standard deviation of γ values in each group of data. n is the total number of γ values, and $\overline{x }$ is the average. The first in-control 20 points of data were used to calculate the LCL values for coalescence and SA processes^29^. For a process to operate in a state of statistical control, the process should be predictable within limits defined by data from the process^29^.

A process capability analysis ensures that a statistical control process is within limits specified by the user. Radiotherapy quality assurance refers to specification limits as action limits, i.e., practices outside the action limits are unsuitable for clinical use^29^. Unlike tolerance limits which serve as warning limits, action limits are used to set a minimum level of process performance. The process capability index, *C*_*pk*_, describes the closeness of the process center to the process target^29^. Here, *C*_*pk*_ was used to assess the potential and performance during the coalescence process, which is calculated as follows^30^

6 $${C}_{pk}=min\left\{{C}_{pl}, {C}_{pu}\right\}=min\left\{\frac{\overline{x }-LAL}{6\left(1-{P}_{x}\right)s},\frac{UAL-\overline{x}}{6{P }_{x}s}\right\},$$

where UAL and LAL are the upper and lower action limits (UAL is 100%), respectively; $\overline{x }$ represents the central line; s is the standard deviation. ${P}_{x}$ is the probability of $x\le \overline{x }$. ${C}_{pl}$ and ${C}_{pu}$ are process capability indices when only one specification exists. Herein, ${C}_{pk}$ is equal to ${C}_{pl}$. LAL, namely action limit, is calculated by^29^

7 $$LAL=T-\frac{\Delta A}{2},$$

8 $$\Delta A=\beta \sqrt{{{\left(\overline{x }-T\right)}^{2}+\sigma }^{2} },$$

where $\Delta A$ is the scope of action limit, namely the difference between UAL and LAL, ${\sigma }^{2}$ is the process variance, and T is the process target value. As in the case of percent γ value, T value is 100%. At a minimum, every institution should document the appropriate β value. It could even be set by expert consensus for different processes^29^. Considering the clinical impact, this study set a value of 4 based on recommended selection principles. After removing the out-control cases, all QA results in each cohort were used to calculate the ${C}_{pk}$ index. In general, the implication of a capability index greater than one for a stable process is that the process is largely operating within specification^28^. Here, a ${C}_{pk}$ value above 1 indicates that the variability of test data, coalesced γ value, was within the inherent variability of process (γ values in SA cohorts).

#### Paired t-test and Wilcoxon signed-rank test

Percent γ values were employed as clinically quality metrics characterizing patient-specific QA procedures, divided into coalescence and SA cohorts. Statistical analyses were executed using GraphPad Prism (version 9.1, La Jolla, CA, USA) and SPSS (version 26.0, Chicago, IL, USA) softwares. To test whether one dataset conformed to normal distribution or not, the Kolmogorov–Smirnov significance hypothesis test (K–S test) and Shapiro–Wilk test were performed for the two cohorts at different tolerance criteria. If one dataset was normally distributed, paired t test was utilized to test whether the average of two paired results was significantly different at the significance level (α) of 0.05. Wilcoxon signed rank test was used for skew distribution data. *P* < 0.05 indicated a statistically significant difference, and Inter Quartile Range (IQR) expressed statistical dispersion.

#### The clinical differences between measured dose-maps vs calculated counterparts

Dose volume histograms (DVH) metrics reconstructed from 2D measurement assembly were compared to TPS dose distributions. The process was carried out by evaluating representative dosimetric parameters from DVHs. D_50_ (dose to 50% volume) was used to indicate the clinically relevant dose discrepancies according to the protocol of ICRU Report 83^31^, for which the absolute percent difference was defined as $\left|\left({D}_{\mathrm{reconstruction}}-{D}_{\mathrm{TPS}}\right)/{D}_{\mathrm{TPS}}\right|\times 100\%$.

Interpolation, namely spatial data interpolation, is needed to estimate the dose value in neighborhood regions of every detector according to the data in known regions and increase the number of data points. In interpolation, the interpolation method is the main determinant of accuracy^32,33^. Reconstruction of voxels in current system was based on a linear interpolation. If there was no measured dose near a reference point, then Verisoft software used the method described by Depuydt et al. to interpolate between adjacent points^33^. Linear interpolation is defined as an interpolation method in which the interpolation function is a polynomial, and the interpolation error on the interpolation node is zero^32^.

#### The creation of error-induced plans

To quantify the sensitivities in identifying clinically relevant systematic and random errors, eight randomly selected plans were modified to obtain the following two types of erroneous plans.

1. Additional dose calculations were run after dosimetric leaf gap (DLG) value was altered by + 1 mm based on the typical value of 1.092 mm established by our radiation oncology community. The error was purposely implemented to examine whether higher spatial resolution could contribute to a more reliably identification on erroneous TPS model. It was hypothesized that all DLG error cases could lead to a lower γ value compared to typical value cases. This implies that an agreement should be mitigated by increasing DLG value, which would be considered “tampering” with the system.
2. A random dose change in machine output was simulated by changing Monitor Units (MU) for each arc of treatment planning. A MU error of + 2% was introduced, and the relative dose weights in each arc of original plan were changed to produce systematic shifts of dose magnitude in the calculated phantom plane.

#### Sensitivity analysis of coalescence and SA procedures in detecting intentional errors

γ index method was used to quantitatively evaluate the sensitivity difference between coalescence and SA cohorts on the induced errors (IE). Dose-maps from coalescence and SA procedures in no-error (NE) plans were compared to those from TPS with induced errors at 3%/2 mm acceptance criteria with global and local normalization. Another hypothesis proposes that the coalescence procedure contributes to an improvement in error-detection ability. Using symbol $\Delta$ to denote the γ magnitude change between NE and IE cases

9 $$\Delta\upgamma ={\upgamma }_{\mathrm{NE}}-{\upgamma }_{\mathrm{IE}}.$$

If $\Delta$ γ > 0, an intentional error is detected; the larger the $\Delta$ γ, the higher the confidence level of identification for error is, and vice versa.

Additionally, a ROC curve was plotted to evaluate sensitivity and specificity in the detection of “abnormal” fluence distributions under various resolution scenarios. To determine the sensitivity and specificity of simulated errors in the QA procedures, the γ values in unmodified cohort served as the control group, and the other error-induced γ values formed the test group. The area under the ROC curve (AUC) and *P* value were combined as performance evaluation index^34^. The more the ROC curve deviates from 45° diagonal, the higher the accuracy of separating an error plan from one without errors for a certain spatial resolution scenario. Conversely, the closer the AUC is to 0.50, the less useful the discriminate ability test is.

## Results

### Coalescence procedure yields higher γ averages and smaller data variability

**Figure 3:**
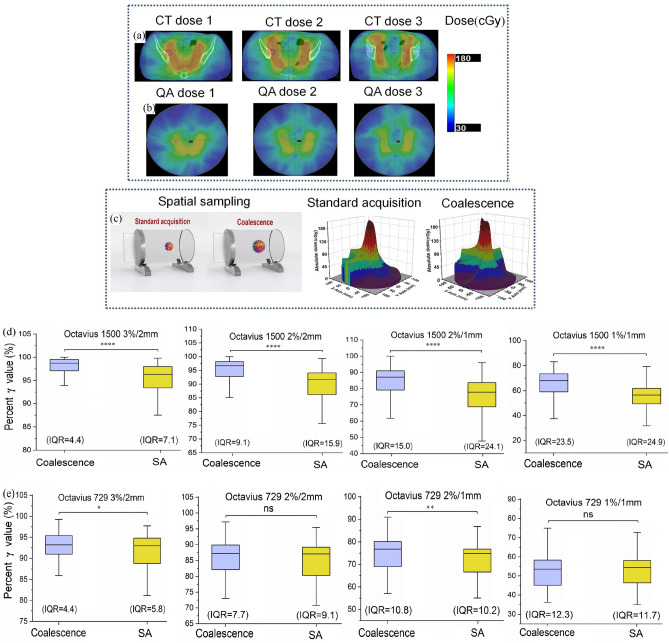
Schematic diagram and percent γ index results in coalescence and SA cohorts. **(a,b)** An example of transverse dose-maps on CT image-based treatment plans and detector-based QA plans correspondingly. Colors indicate dose magnitude. **(c)** Coalescence procedure yields double spatial sampling points compared with standard acquisition (left figure). **(d,e)** The γ results in coalescence versus SA cohorts at different acceptance criteria. As showed by box plots, centerlines show the medians, box limits indicate the 25th and 75th percentiles, and whiskers extend to the minimum and maximum. If there were statistically significant differences between coalescence and SA cohorts, IQR in coalescence cohorts was lower than in SA cohorts. Asterisk indicated the statistically significance at a 5% level (two-tailed). ****P < 0.0001, ***P < 0.001, **P < 0.01, *P < 0.05, ns: *P* > 0.05.

To understand the effects of different spatial resolution and sampling points on QA results, we calculated γ values for each cohort. On one CT image-based pelvic treatment plan, three transverse dose-maps are shown in Fig. 3a, and corresponding detector-based dose distributions in cylindrical phantom are illustrated in Fig. 3b, respectively. Each color represents a different magnitude of dose for one fraction. The schematic diagram in Fig. 3c shows a coalescence procedure that produces double spatial sampling points compared to standard acquisition, and 3D dose distribution is reconstructed accordingly (right figure). Generally, it is necessary to interpolate the region where the ion chamber does not exist to increase evaluation points and acquire delivery dose within 3D volume. By analyzing γ values calculated on interpolated data points, our results indicated that detector 1500 showed higher averages in coalescence cohorts at different criteria and the dispersion degree was spread out smaller (Fig. 3d). This implied that the accuracy of the interpolation algorithm depends on the uncertainty of the measured plane dose. Coalescence increases the number of measurement points resulting in higher planar dose accuracy. Therefore, there may be fewer errors during interpolation process. These lower IQR results in coalescence cohorts indicated that the γ values distribution had less variability around average (Fig. 3d). Accordingly, the probability of outliers occurring in coalescence cohorts is lower, and the sample data provided by coalescence can better predict information about the population. Nevertheless, detector 729 yielded results that showed less significant γ value discrepancies between both cohorts, with the average in coalescence cohorts slightly higher than in SA cohorts (Fig. 3e). For the 2%/1 mm and 1%/1 mm criteria, coalescence cohorts showed slightly lower consistency than SA. As shown in Fig. 3e, the averages in both cohorts were less than 95% at 3%/2 mm, commonly used acceptance criteria in the radiotherapy community.

### DVHs reconstruction

**Figure 4:**
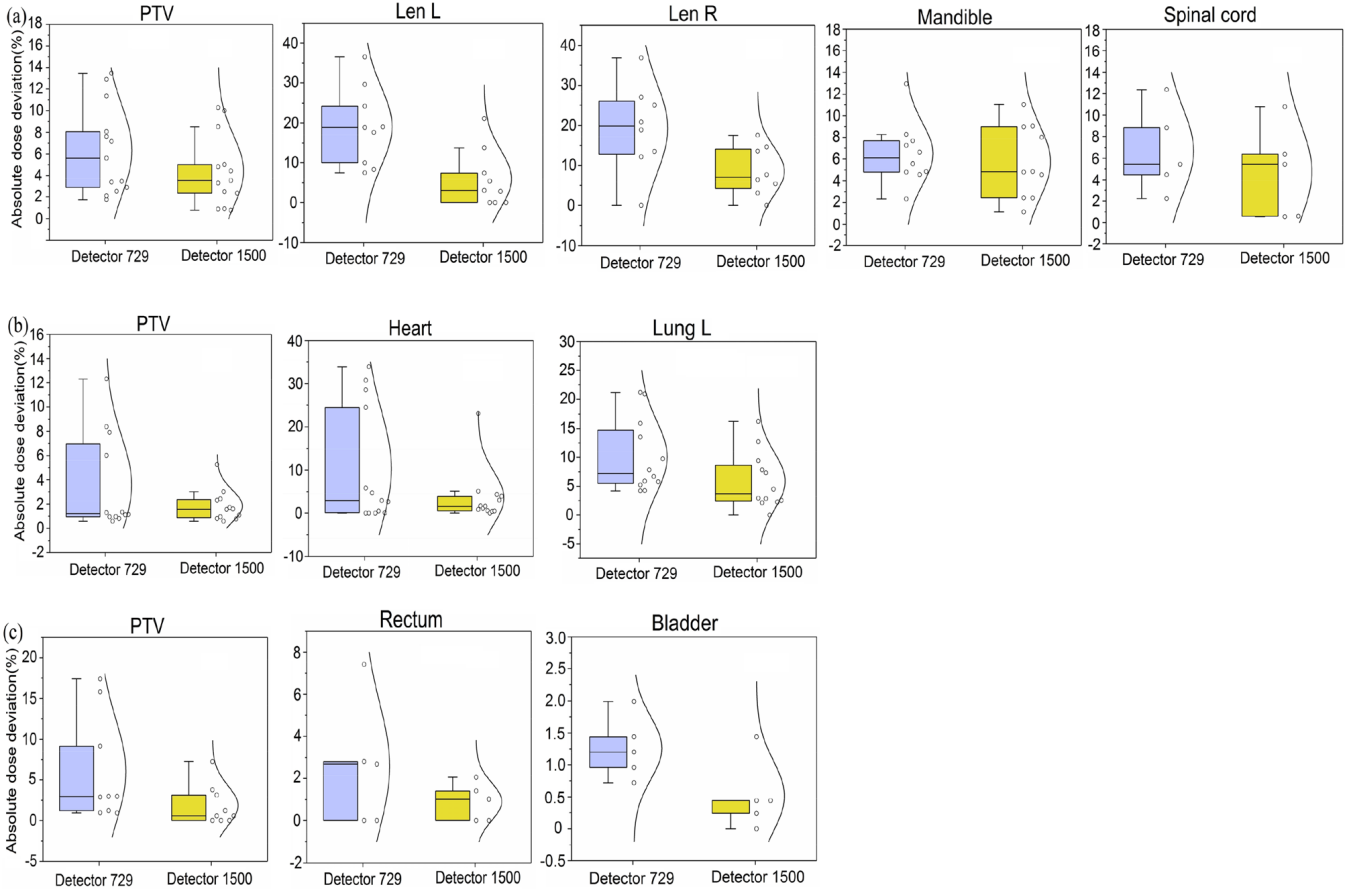
Absolute percent dose deviations in PTVs and partial OARs. **(a)** PTVs, left lens, right lens, left parotid gland, mandible, and spinal cord in head & neck cases. **(b)** PTVs, heart, and left lung in thorax cases. **(c)** PTVs, urinary bladder, and rectum in pelvic cases. The differences between individual values in 1500 cohorts were smaller than those in 729 cohorts, as shown in the boxplot and the points and lines of normal distribution.

The calculation is determined for structures located completely inside the measuring range of detector (27 × 27 cm^2^). No dose information from TPS is needed for anatomy-based dosimetric metrics, and the reconstruction process is also independent of γ values. As this software prevents DVHs reconstruction based on coalescence dose-maps, we used SA dose-maps in both detectors to compare the effects of detector planes with different spatial resolutions on DVH reconstruction accuracy. The box plots for D_50_ in PTVs and OARs revealed that the reconstruction dose performed by detector 1500 established a better agreement with TPS-predicted ones than detector 729 did. Apart from that, there was a smaller data spread out (Fig. 4a–c), which suggested that an enhancement of detector resolution might conducive to an improvement of reconstruction accuracy in DVHs. To create volume dose distribution, Verisoft software used a linear interpolation algorithm, which identifies patterns in known data sets (e.g., a series of discrete measured points). Then, those patterns are used to create numerical estimates of points that have not yet been recorded. Its main application is to reasonably compensate for the missing data. Therefore, this may be explained by the fact that detector resolution is higher, more data points are measured, and fewer points are estimated by interpolation, consequently, the introduced dose estimation error is less. For the same detector, reconstruction accuracy in abdominal tumors was better than those in head and neck and thorax tumors, in which thorax tumors yielded the worst precision. Linear interpolation algorithm is based on an assumption that the change and development of the phenomenon are linear and uniform. Compared to abdominal structures, thorax structures contain lung tissue whose mass density is close to air and less than water. Therefore, the better accuracy in reconstructed dose might be a consequence of more homogeneous patient structures of interest, which is dependent upon the type of interpolation used.

### Sensitivity and performance in various resolutions based on γ value difference and ROC analysis

**Figure 5:**
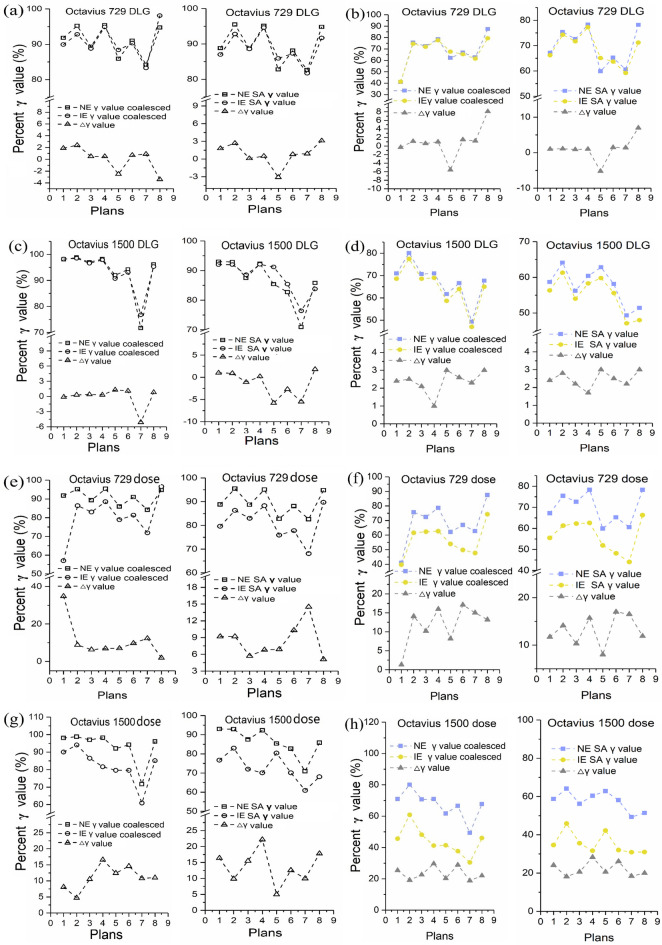

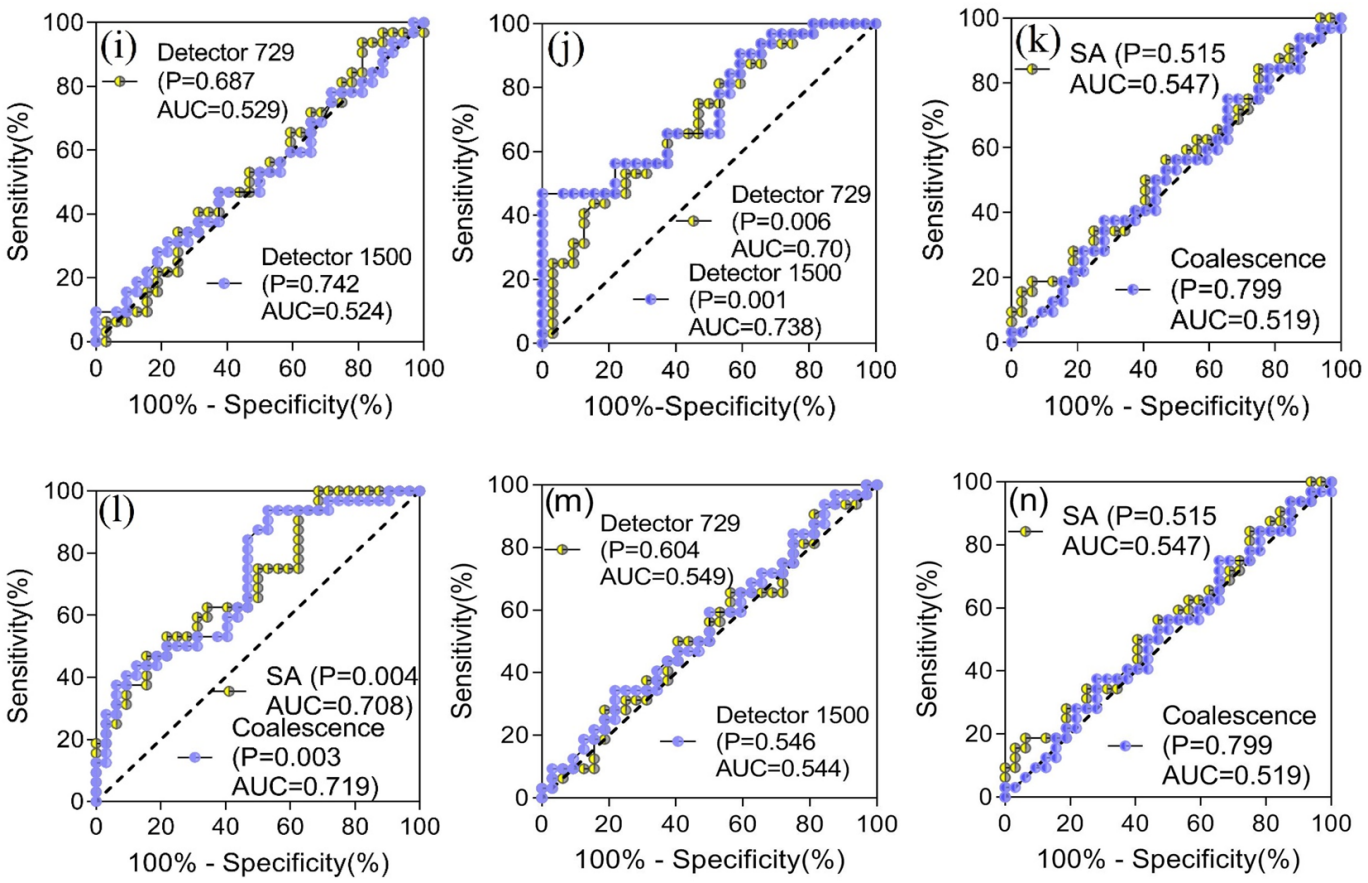
Sensitivity comparisons in detecting DLG and dose errors in various resolution scenarios. **(a–h)** DLG and dose errors detected by both detectors in coalescence and SA cohorts with global (**a,c,e,g**) and local (**b,d,f,h**) normalization. Comparative ROC curves: **(i–j)** DLG and dose errors detected by both detectors using mixed γ values in coalescence and SA cohorts; **(k–l)** DLG and dose errors detected by coalescence and SA procedures using γ values in both detectors; (**m,n**) DLG errors detected by both detectors and coalescence as well as SA cohorts using 1%/1 mm criteria (global normalization). Curves along the diagonal with an AUC of 0.5 indicate a test whose outcome is not significantly different from a random guess.

Subsequently, we investigated the sensitivities in various spatial resolutions on detecting systematic model error and random dose error, i.e., artificially introduced different errors. The γ values and its difference between no-error and induced-error cases for each of the eight VMAT cases in coalescence and SA cohorts in both detectors, at 3%/2 mm criteria with global vs local normalization, are displayed in Fig. 5a–h (Fig. 5a,c,e,g showed results of global normalization, Fig. 5b,d,f,h showed results of local normalization). Additionally, ROC curve was used to further assess sensitivity and specificity in separating IE cases from NE cases in various resolution scenarios. A similar trend was detected for DLG and MU errors in that the sensitivity in coalescence cohorts was not higher than that in SA cohorts. For DLG error, there was case-specific different sensitivity, namely, mixed higher ($\Delta$ γ < 0) and lower ($\Delta$ γ > 0) γ values in IE cases compared to those in NE cases, as shown in Fig. 5a–d. Consequently, ion chamber detectors were unable to accurately separate DLG error of 1 mm regardless of coalescence or SA procedures, while areas under ROC curve were slightly greater than 0.5 (Fig. 5i,k). To further verify this result, we have performed the experiments again using the strictest criteria of 1%/1 mm (global normalization) as suggested by TG 218 guideline. As a result, the resuts of most stringent tolerance criteria were not significantly different from the 3%/2 mm tolerance criteria (Fig. 5m,n). This contradicts the hypothesis that all DLG-error cases may lead to a lower γ value. Dose deviations may occur at any time during the calculation and delivery process. The results showed that regardless of global or local normalization, γ values in IE cases were lower compared to NE cases in coalescence and SA cohorts. Dose error sensitivity in two spatial sampling scenarios showed slightly different behaviors, and the potential effects of spatial resolution on γ value varied from case to case. Higher resolution cohorts had slightly greater areas under ROC curve than those in lower resolution cohorts (Fig. 5j,l). However, higher resolution cohorts showed a greater statistical difference between NE and IE cases (*P* = 0.001, 0.003, 0.022 vs *P* = 0.006, 0.004, 0.055). Therefore, the potential for improving the detectability of dose error was detected by increasing spatial resolution and sampling points in coalescence cohorts.

### Increased tolerance limits and improved process capability for higher spatial resolution scenarios

**Figure 6:**
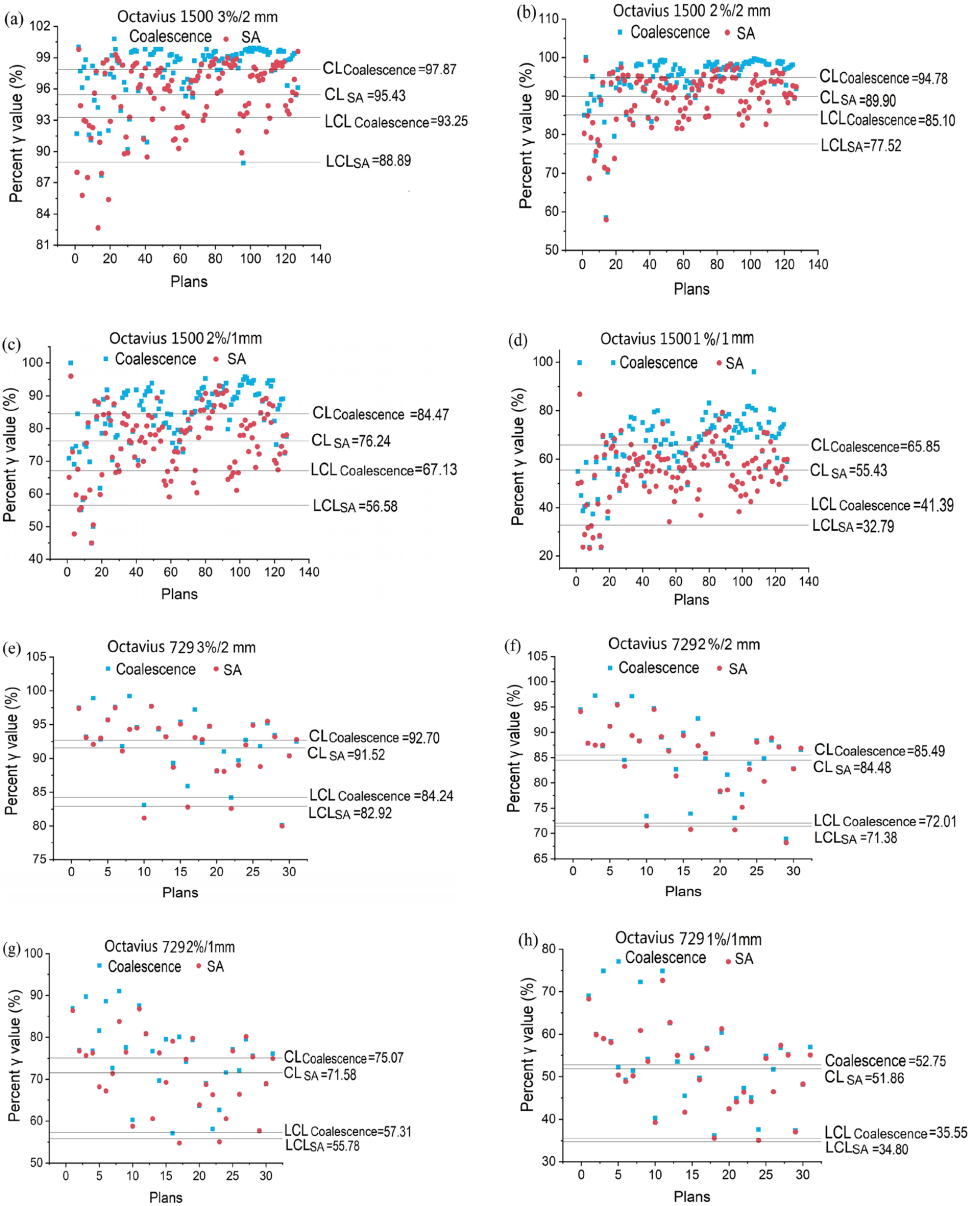
Individual control charts of percent γ values in VMAT QA processes at four acceptance criteria. The central line was represented by the average of a group of data. **(a–h)** γ values in coalescence and SA cohorts for both detectors, respectively. The arbitrary plan number for each dose-map was listed on x-axis. The differences between CL and LCL in coalesced scenarios were smaller than in SA scenarios. *CL* center line, *LCL* lower control limit.

Apart from directly comparing QA results, we used SPC and PCA methods to demonstrate a statistical expectation of the different performances between coalescence and SA cohorts. LCL values were calculated for each cohort. Between the two cohorts, the LCL difference ranged from 5.36 to 10.55% for detector 1500 and 0.37% to 2.25% for detector 729 (Table 3). In addition, based on LAL values calculated from $\Delta A$ in SA cohorts (Table 1), ${C}_{pk}$ index values were calculated to assess the potential and performance during coalescence (Tables 2, 3). Table 2 showed that detector 729 yielded a lower process capability of 0.79, 0.76, 1.10, and 1.34, but detector 1500 exhibited somewhat different results of 0.94, 1.42, 1.19, and 1.60 in magnitude. In order to improve processing capability, it appears that reducing moving ranges (data dispersion) could be beneficial (Table S1). The higher LCL and ${C}_{pk}$ index values in 1500 coalescence could be attributed to the fact that higher γ values obtained from all MLC leafs bank and tongue-and-groove being sampled along a longitudinal direction. Next, SPC individual control charts were drawn to monitor patient-specific QA processes in coalescence and SA procedures (Fig. 6). The tolerance limits (LCL) calculated for detector 1500 in coalescence and SA cohorts were 93.25% and 88.89% (3%/2 mm criteria, Fig. 6a), 85.10% and 77.52% (2%/2 mm criteria, Fig. 6b), 67.13% and 56.58% (2%/1 mm criteria, Fig. 6c), and 41.39% and 32.79% (1%/1 mm criteria, Fig. 6d), respectively. Further, the cases/proportion lower than the tolerance limits were 11/8.73%, 10/7.93%, 5/3.97%, and 8/6.34% in coalescence cohorts, 6/4.76%, 8/6.34%, 5/3.97%, and 8/6.34% in SA cohorts, respectively. For detector 729, the tolerance limits were 84.24% and 82.92% (3%/2 mm criteria, Fig. 6e), 72.01% and 71.38% (2%/2 mm criteria, Fig. 6f), 57.31% and 55.78% (2%/1 mm criteria, Fig. 6g), and 35.55% and 34.48% (1%/1 mm criteria, Fig. 6h), respectively. In addition, the cases/proportion lower than the tolerance limit were 2/6.45%, 1/3.23%, 0/0%, and 0/0% in coalescence cohorts, as well as 4/12.90%, 3/9.68%, 2/6.45%, and 0/0% in SA cohorts, respectively.

**Table 1:** Difference between upper action limit (UAL) and lower action limit (LAL) in both detectors.

Tolerance criteria	$\Delta A$_Detector729 coalescence_ (%)	$\Delta A$_Detector729 SA_ (%)	$\Delta A$_Detector1500 coalescence_ (%)	$\Delta A$_Detector1500 SA_ (%)
3%/2 mm	33.74	38.02	12.57	22.47
2%/2 mm	64.86	67.38	28.46	47.04
2%/1 mm	106.10	117.99	71.14	102.80
1%/1 mm	193.00	195.56	125.16	183.93

**Table 2:** Action limits (LAL) of detectors 729 and 1500.

Tolerance criteria	LAL_Detector729 coalescence_ (%)	LAL_Detector729 SA_ (%)	LAL_Detector1500 coalescence_ (%)	LAL_Detector1500 SA_ (%)	UAL (%)
3%/2 mm	83.13	80.99	93.58	88.77	100
2%/2 mm	67.57	66.31	85.80	76.48	100
2%/1 mm	46.93	41.00	64.43	48.60	100
1%/1 mm	3.40	2.22	37.47	8.03	100

**Table 3:** Lower control limits (tolerance limits) in each cohort and process capability index values.

Tolerance criteria criteria	LCL_Detector729 coalescence_ (%) (%)	LCL_Detector729SA_ (%)	LCL_Detector1500 coalescence_ (%)	LCL_Detector1500 SA_ (%)	${C}_{pk}$(_729_)	${C}_{pk}$(_1500_) _coalescence_)
3%/2 mm	84.24	82.92	94.25	88.89	0.79	0.94
2%/2 mm	71.38	71.01	86.10	77.52	0.76	1.42
2%/1 mm	57.31	55.78	67.13	56.58	1.10	1.19
1%/1 mm	34.80	32.55	41.39	32.79	1.34	1.60

*LCL* lower control limit, ${C}_{pk}$ process capability index.

### Increased sampling points lead to better profile accordance and smaller γ values

**Figure 7:**
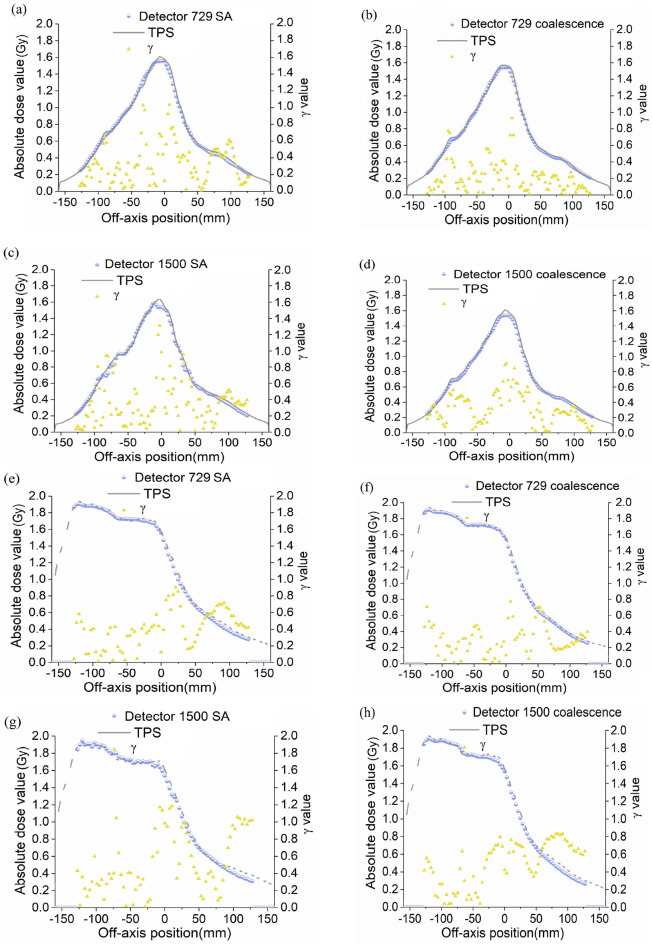
Dose profile comparison of transverse view between measured and calculated dose distribution in an abdominal 6 MV VMAT plan. **(a–d)** Dosimetric profile representations of SA and coalescence results along the lateral left and right directions in detectors. **(e–h)** Dosimetric profile representations of SA and coalescence results along the positive slope diagonal (from the upper left to the lower right) in detectors.

Finally, the measured and calculated profiles were compared as well as absolute γ values at different off-axis positions, which provided a more intuitive way to evaluate QA results in coalescence and SA procedures. Figure 7a–h showed the dosimetric profile analysis of a transverse slice of coalescence and SA for a pelvic case, which were acquired at 3%/2 mm criteria. After comparing measured and calculated profiles, a little different agreements were found within the radiation field, penumbra, or steep dose gradient regions, in which coalescence (Fig. 7b,d,f,h) exhibited better agreement than SA (Fig. 7a,c,e,g). A steep dose gradient could result in fewer pixels passing the tolerance criteria. By combining the results of the first section, we concluded that coalescence could provide a more accurate dose estimation in steep dose gradient regions. The absolute γ index values at each position were displayed by selecting the absolute dose values check box, and a minimum γ value was searched within a search distance of 2 mm. We found that some points with a γ value greater than 1 appeared in SA profile comparison (Fig. 7a,c,g), however, they passed the coalescence comparisons (Fig. 7b,d,h), with the absolute γ values less than 1 at corresponding positions. Coalescence yielded relatively smaller γ values at more positions. Specifically, γ value is calculated as a dimensionless metric based on finding the shortest Euclidean distance for each reference point. For the acceptance criteria of the dose difference 3% and the distance to agreement of 2 mm, the 2 mm and 3% formed an ellipsoid around the reference point. The reference point passed if an evaluated point was located within this since γ value would be < 1. For each reference point at off-axis positions, γ values were calculated individually in the evaluated distribution. All evaluated points were added together to find a minimum, which was referred to as the final γ result. The smaller the absolute γ value, the lesser may be the distance and/or the dose difference between the reference and evaluated points. Consequently, increased evaluation points in coalescence procedure resulted in a greater probability of getting smaller γ values.

## Discussion

The 2D detectors used in clinical practice to detect dosimetric differences between calculation and delivery require that the measured dose conform to the Nyquist sampling theorem. Therefore, patient specific QA should comply with two criteria^35^. First, the size of a single detector should ensure the detectability of sufficiently small misalignments between measured and calculated values. For 2D detectors with single detector diameters in the range of several mm, this implies that the influence of the measuring process, i.e., the lateral response function of a single detector, needs to be quantitatively accounted for. In regions between adjacent detectors where the lateral response function is zero, whether the measurement sensitivity may be affected by MLC misalignments or dose deviations, the problem is thereby transferred to the appropriate spacing between single detectors, i.e., the second criterion. To achieve the second criterion, a method that coalesce two adjacent measurements to increase sampling points and spatial resolution was developed. The leaf width of our linear accelerator determined that one-half of leafs and one-third of the tongue-and-grooves were sampled during one single measurement using detector 729 when the collimator angle was 0°. Coalescence in a longitudinal direction allowed all leafs to be sampled, thus doubling the sampling number of tongue-and-grooves. For VMAT plans, the collimator angle is not generally set to 0°, and single ion chamber sample less-complete leaf and tongue-and-groove during each measurement, but their total number of samples remains the same as 0°. For detector 1500, most leafs and tongue-and-grooves were acquired during one single measurement regardless of whether the collimator was 0°. Consequently, all of them were sampled during coalescence procedure, and the acquisition accuracy of ion chamber on leafs were further improved. In detector 729, the relatively coarse spatial resolution and sparse sampling may increase measurement uncertainty when used to reconstruct 3D dose and lead to more uncertainties with the γ index calculation than higher resolution detectors such as 1500 and 1000^SRS^^16,36–38^. Therefore, we speculate that the potential errors in the detector 1500 coalescence process might be fewer than those in detector 729. These may account for significant differences in γ values between coalescence and SA cohorts for detector1500 at four tolerance criteria, whereas detector 729 yielded statistically significant results only at 3%/2 mm and 2%/1 mm criteria (*P* = 0.034, *P* = 0.004). Combined with the sampling analysis of ion chamber detector, there may be discrepancies in QA results across diverse spatial resolution scenarios due to MLC leaf width, single detector size, and the spacing between adjacent detectors.

The spatial sampling frequency (minimum sampling rate) of a detector must comply with Nyquist sampling theorem to detect minimum fluence deviations that might occur during the dose calculation and delivery^35^. The fill factor, i.e., the fraction of total detector area covered with sensitive areas of detectors, is a crucial characteristic in detectors, as well as the resolution and sampling frequency^7,39^. The fill factor is determined by the full width at half maximum (FWHM) of lateral fluence response function in a single detector and the cell area attributed to each detector^7^. Its significance was demonstrated when the sensitivity of the diode detector to fluence perturbations caused by misaligned MLC leafs was slightly lower than that of the ion chamber detector, due to the higher filling factor in the ion chamber detector^40^. Detector 1500 had a fill factor of 0.86 in a single measurement, and after coalescing two adjacent measurements, 50% of the response contours overlapped to produce 1.00^7,12,40^. For a single measurement by detector 729, a fill factor of 0.5 was obtained, which failed to achieve 1.00 by coalescing two measurements^7,12,40^. With a dose error of + 2%, some cases in coalescence cohorts achieved higher error sensitivity while others showed the reverse compared to SA ones. Using the γ analysis alone, it is difficult to quantify the sensitivity and specificity of a test to detect abnormal fluence distributions^34^. Therefore, the sensitivity and performance in different resolution scenarios were assessed with a ROC curve. AUC summarizes the discriminate ability in various spatial resolutions between those with the simulated errors and original unmodified cases^41–43^. The results indicated that the AUC values in higher resolution scenarios were slightly larger than those in lower ones (Fig. 5j,l,m,n) and the γ value difference was statistically significantly larger in higher resolution scenarios. Consequently, the sensitivity of an ion chamber detector to dose error was inferred to depend on the magnitude of the fill factor. Increasing the spatial resolution contribute to an increased sensitivity to induced dose error. In contrast, there was no statistically significant difference between IE and NE cases for DLG error (*P* > 0.05, Fig. 5i,k), and AUC values in higher resolution scenarios were slightly lower than those in lower ones (Fig. 5i,k). Similar to other studies^44–47^, these results indicated that the atypical or erroneous parameter value in MLC modeling failed to be detected reliably given our current patient-specific QA protocols. Systematic errors that go undetected are contrary to the basic tenets of quality assurance as enumerated in the Introduction section. To resolve the issue of insufficient system error sensitivity, machine learning has the potential to be an effective solution. More recently, the use of deep learning and radiomics methods to detect various errors from TPS modeling and/or dose delivery process is superior to traditional physical measurements^48–51^.

TG-218 recommended and supported by previous studies^21^, the use of SPC methods to set process-based tolerances and action limits could account for all aspects of variations in IMRT/VMAT quality assurance^52–29^. SPC enhanced its effectiveness in monitoring patient specific QA process with the general purpose of identifying factors affecting process out-of-control^6,21^, which was used in our study to examine whether tolerance and action limits differed between coalescence and SA. The tolerance limit is a warning limit that indicates a process is changing and needs to be addressed when it exceeds the tolerance limit^26^. As control chart limits are designed to characterize process performance, they are ideal for defining tolerance limits^26^. Processes with more variability may have correspondingly wider control limits. Once the control limit is set, it can be monitored for changes to the process^26^. Generally, tolerance limits are dependent only on the process, i.e., the process data determines tolerance limits. Nevertheless, action limits are solely determined by certain clinical requirements independent of tolerance limits. Furthermore, even within the same radiotherapy community, tolerance limits would probably vary for similar processes^26^. LCL values (tolerance limits) determined in coalescence cohorts at four criteria for both detectors were higher than in SA cohorts. While monitoring patient-specific QA processes, SPC individual control chart showed that cases in coalescence cohorts with γ values lowering its LCL were greater than those in SA cohorts for detector 1500 (Fig. 6a–d). These results indicated that coalescence procedure could detect more potential failure QA results than SA while enhancing action thresholds, thereby making the QA process more secure. Notably, detector 729 had lower tolerance and action limits than detector 1500. The process capability index ${C}_{pk}$ can be used to measure process potential and performance when a process is in the state of “statistical control”^6^: the higher the ${C}_{pk}$ value, the more capable the process^21^. Essentially, a higher ${C}_{pk}$ value indicates that the machine completes its patient specific QA process at a higher process yield. We used ${C}_{pk}$ index to describe the number of γ values in coalescence cohorts that were contained within the variability of the SA γ values. Assuming that the coalesced γ values are Gaussian distributed, a ${C}_{pk}$ value that is > 1 for one specification indicates that almost all of coalescence-yielded γ values are above the LCL value in SA procedure at corresponding criteria. Compared to SA, detector 1500 coalescence demonstrated a smaller variability in which ${C}_{pk}$ values were above 1.0. It means that the coalescence procedure in detector 1500 could provide more robust and reliable QA results. Even though ${C}_{pk}$ value at 3%/2 mm criteria was 0.94 but close to 1. ${C}_{pk}$ values in detector 729 were weaker indicators that γ values coalesced were likely above the tolerance limits in SA cohorts. The ${C}_{pk}$ values of detector 1500 were higher than those of detector 729, which indicated that detector 1500 coalescence procedure exhibits a better process capability, while the coalescence procedure of detector 729 needs improvement. Accordingly, there is reasonable statistical confidence that coalescence approach can provide a higher tolerance level in patient specific QA procedure of VMAT techniques examined than SA. These findings suggested that establishing reliable tolerance and action limits for patient-specific QA is challenging if doses are acquired using a detector that has a lower data acquisition density^36^. Notably, the cases with γ values below the LCL in coalescence cohorts were not always more than in SA cohorts for detector 729. The lack of accuracy in 3D dose reconstruction by linear interpolation algorithm under lower spatial sampling scenarios and the minuscule LCL value difference between coalescence and SA cohorts in detector 729 might both contribute to the phenomenon. 126 dose-maps at positions of isocenter and 5 mm-couch shift were obtained for detector 729, respectively. However, most dose-maps failed be coalesced because of the errors in overlapping dose difference. These results indicated that lower spatial resolution and fewer measurement points might lead to more errors and contribute to a larger measurement uncertainty range that occurred at the first data acquisition stage. In fact, the process control charts could be implemented without a large database. They can be created with only 20–30 data points^6,23^. In addition, the magnitude in ${C}_{pk}$ index was detected to be proportional to the distance between LCL and CL. Due to an increased standard deviation estimate, a larger distance would usually reduce the process capability^25^.

At first glance, our results may seem unsurprising, and multiple previous studies have likewise noted similar coalescence procedure in ion chamber detectors^4,5,7,9–11^. However, the current work is unique in that the SPC technique and PCA herein are used as a more reliable argumentation on the coalescence process in meliorating spatial resolution and sampling frequency. To the best of our knowledge, no other methods of resolution improvement in ion chamber detectors have been reported. Our results implied that the coalescence-based procedure may contribute to more accurate acquirement in and out of radiation fields by increasing the sampling resolution without additional material.

## Conclusion

Implementing the coalescence sequence increases the number of sampling points and eliminates gaps between adjacent detectors, therefore, it leads to improvements in patient-specific QA process. This study provides new insights into the sampling delivery dose and error detectability, further broadening the current understanding on QA results. The type of interpolation algorithm used may contribute to a difference in dose reconstruction uncertainties in patient structures of interest with various physical density ranges. We also uncover that the magnitude of filling factor in ion chamber detector determine its sensitivity to simulated dose error.

## Supplementary Information

Supplementary Information.

## Data Availability

All data generated and analyzed during this study are included in the article and Supplementary Materials.
