# Supplementary material for: Correlation between patient-specific quality assurance in volumetric modulated arc therapy and 2D dose image features
Source: Sci Rep. 2023 Mar 10;13:4051. doi: 10.1038/s41598-023-30719-4 (PMC10006091; doi:10.1038/s41598-023-30719-4)
Supplement: Supplementary file 1 — Supplementary Information. [file 41598_2023_30719_MOESM1_ESM.docx]

**Supplementary materials**

Figure S1. Representations of tumors and organs at risk as well as the path of conical beams.


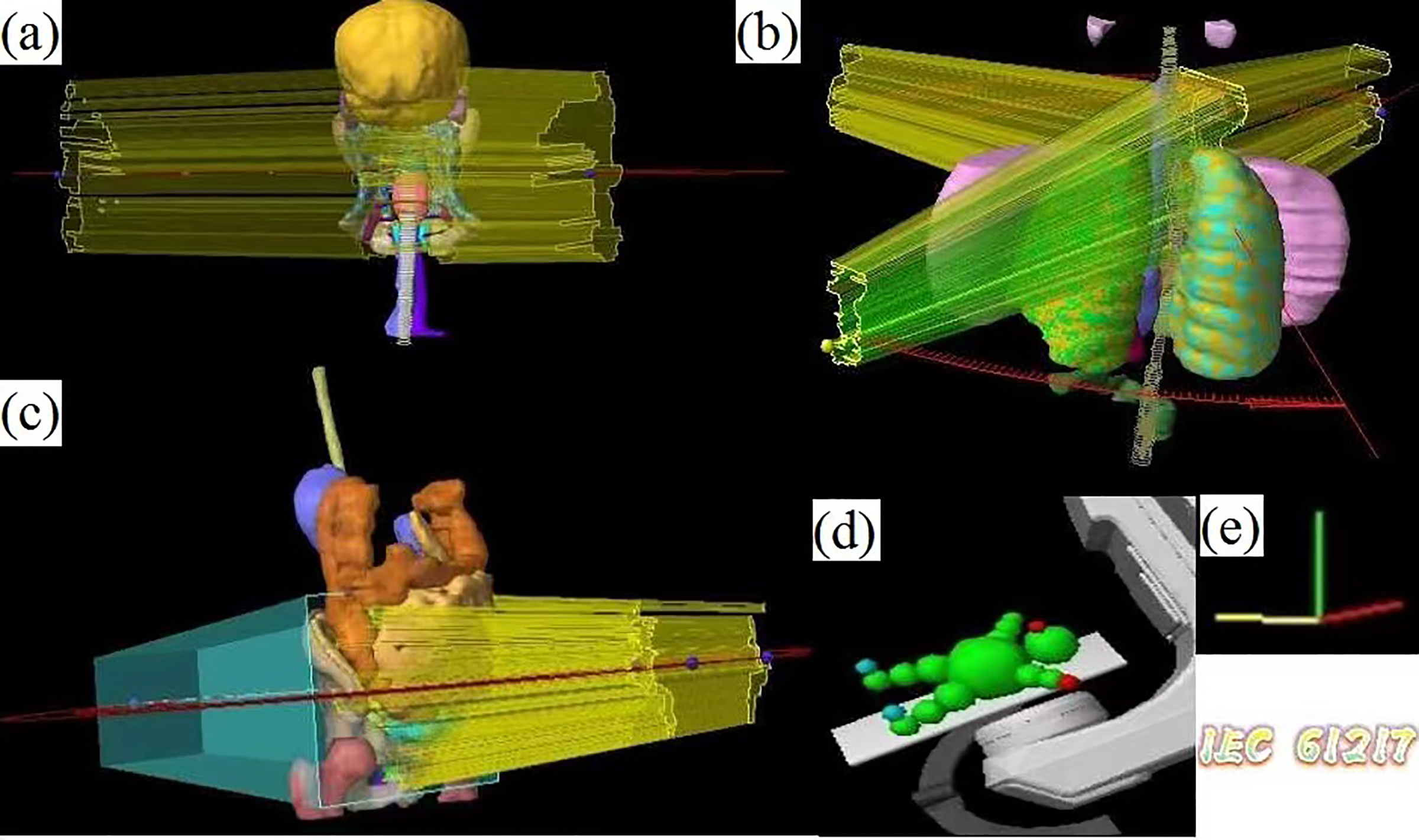


Representatives of tumors and organs at risk as well as the path of conical beams. (a) A head & neck case; (b) A thorax case; (c) A pelvic case; (d-e) Radiotherapy equipment uses IEC 61217 coordinates.

Figure S2. Heat map demonstration of γ values.


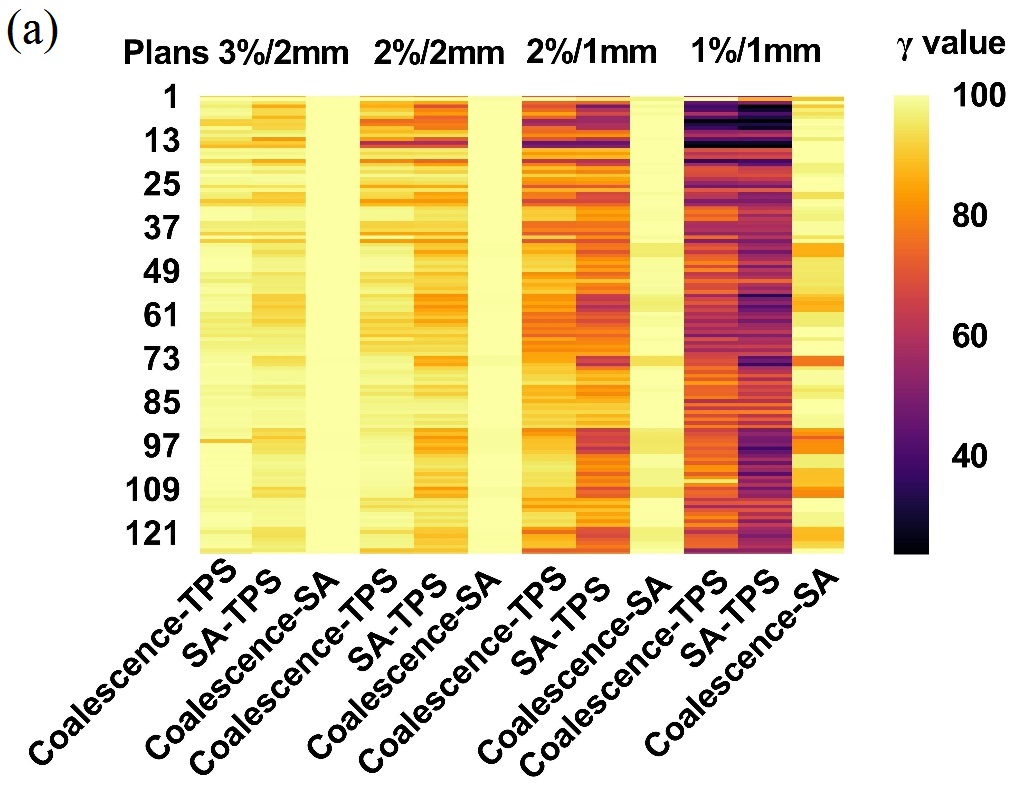

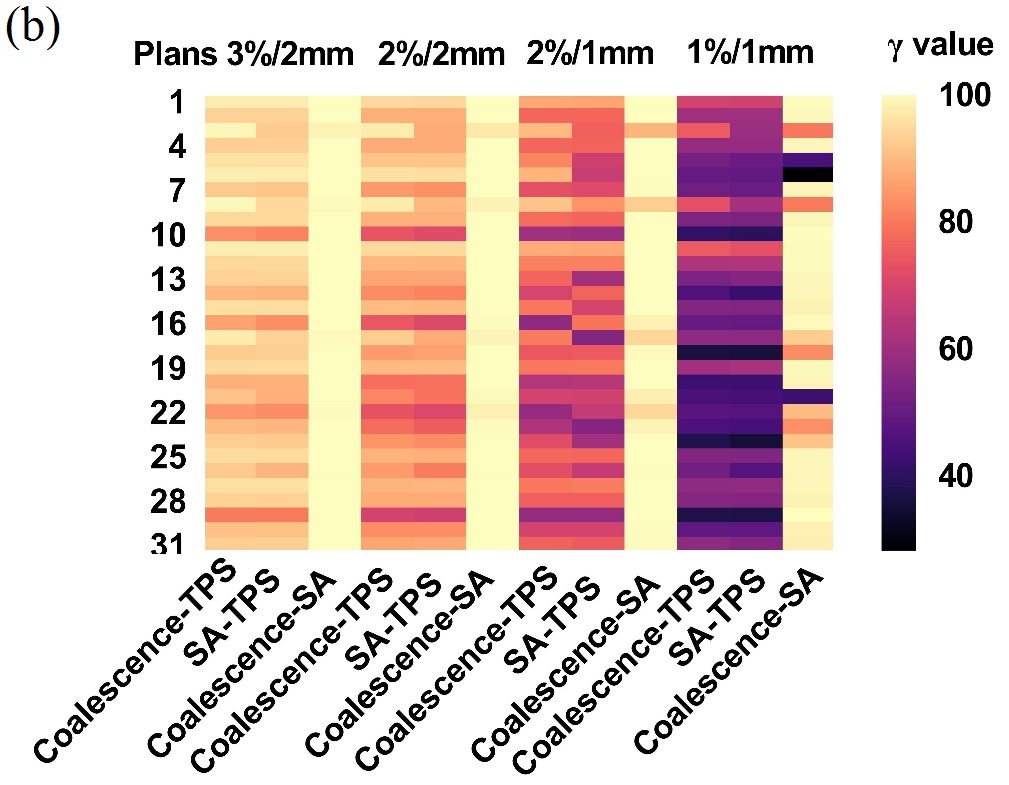


Heat map demonstrations of γ results in coalescence versus TPS, SA versus TPS, and coalescence versus SA at different acceptance criteria using detectors 1500 and 729, respectively.

Figure S3. Normal probability plots of γ values in coalescence and SA cohorts for both detectors.


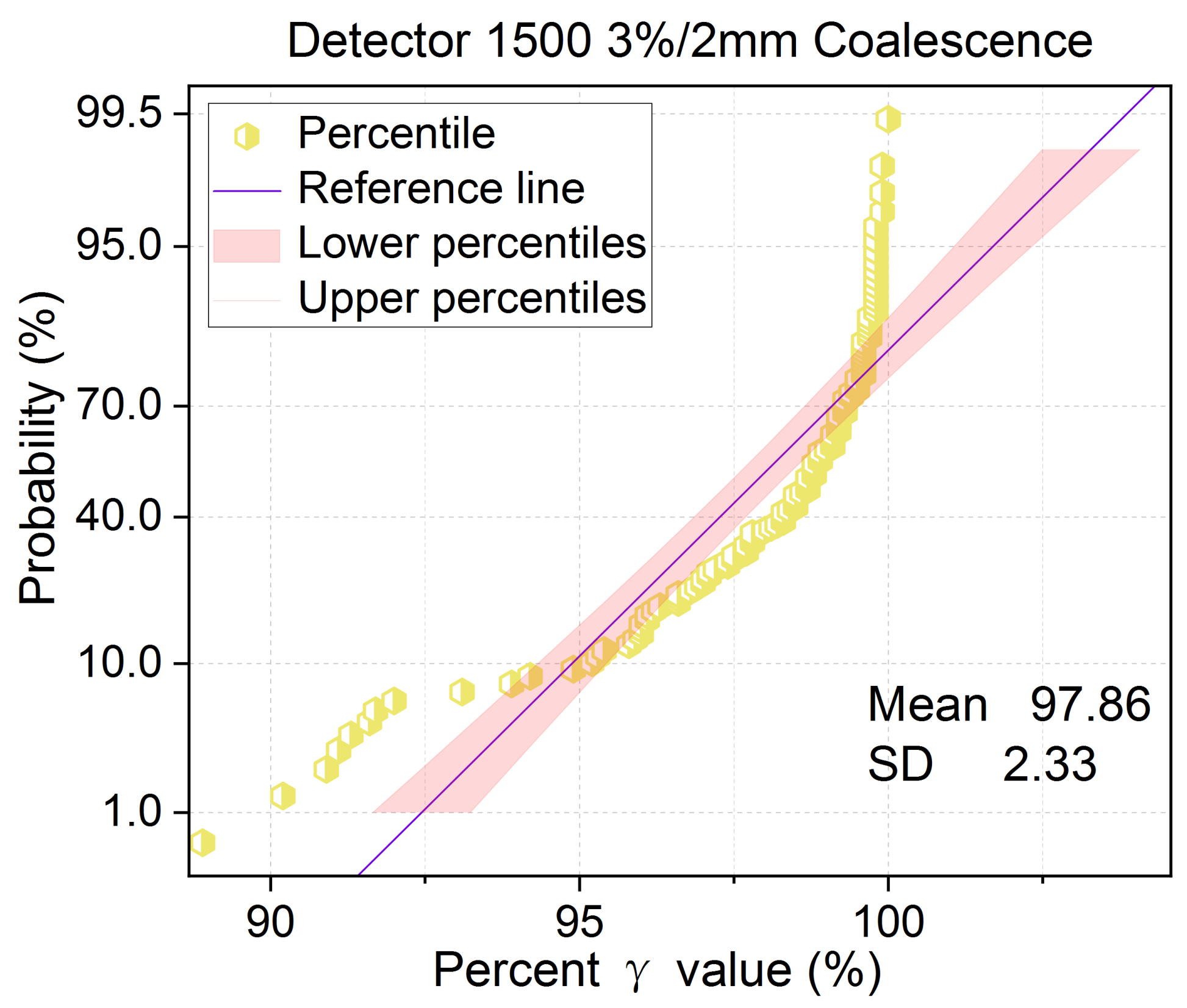

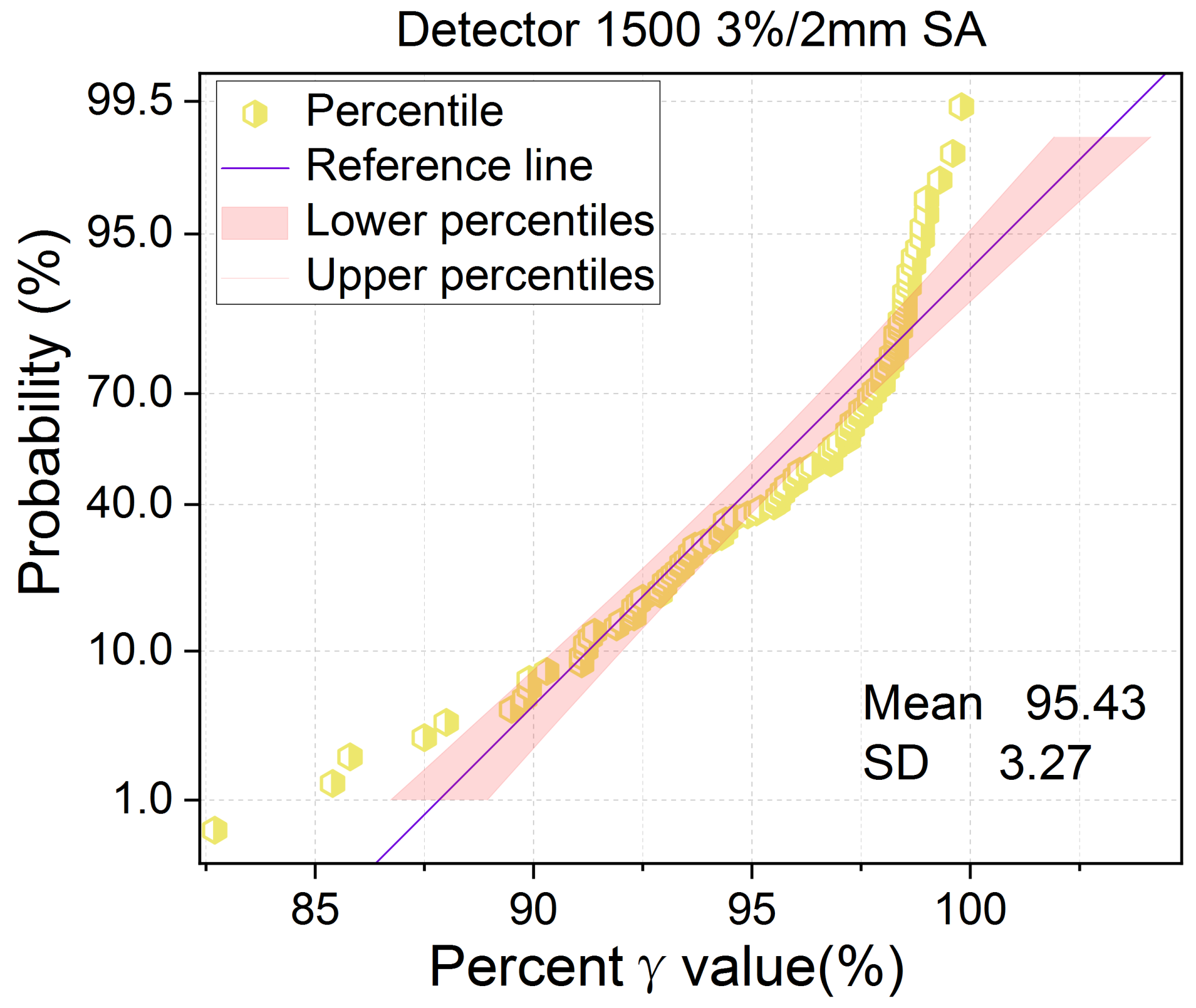

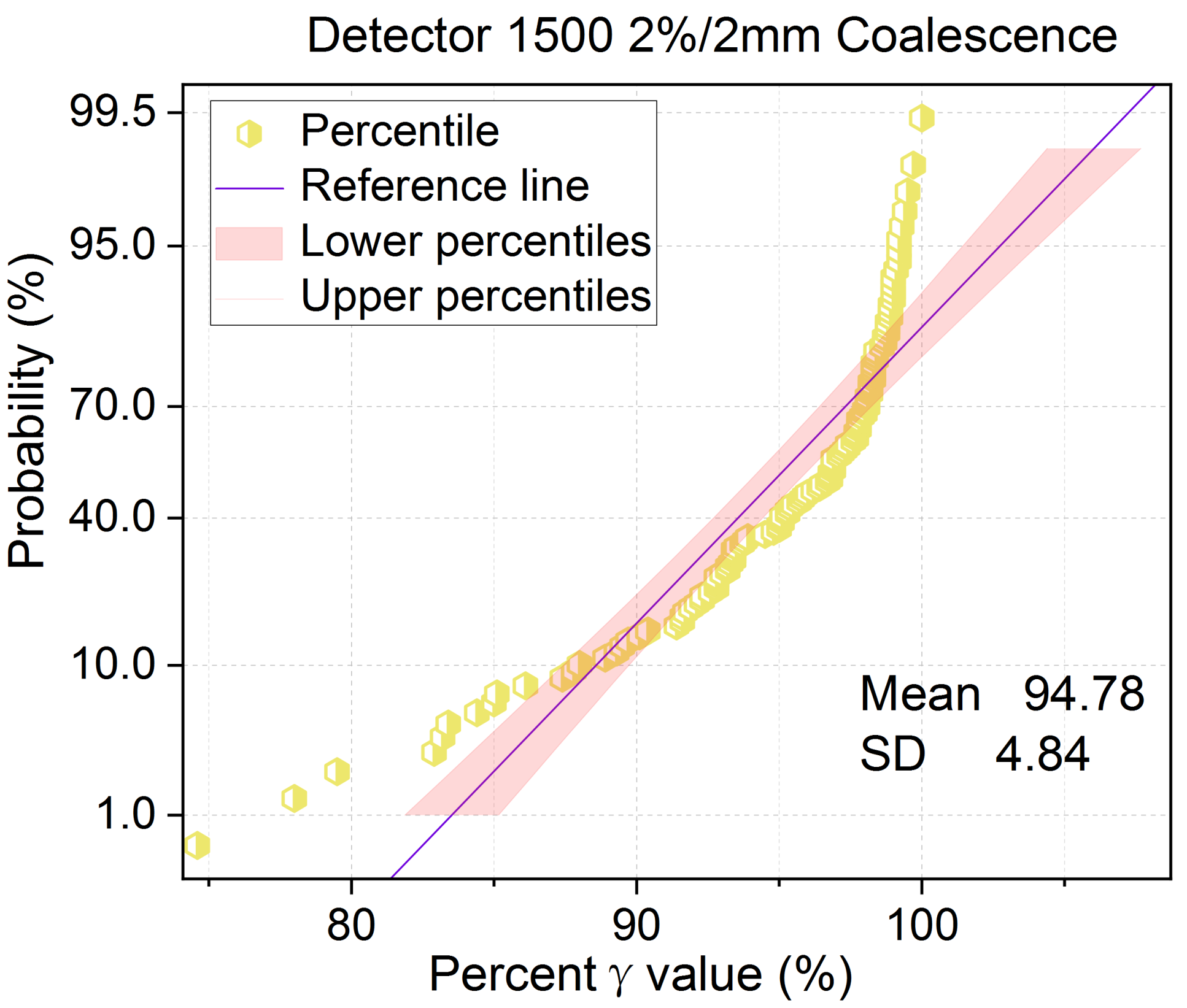

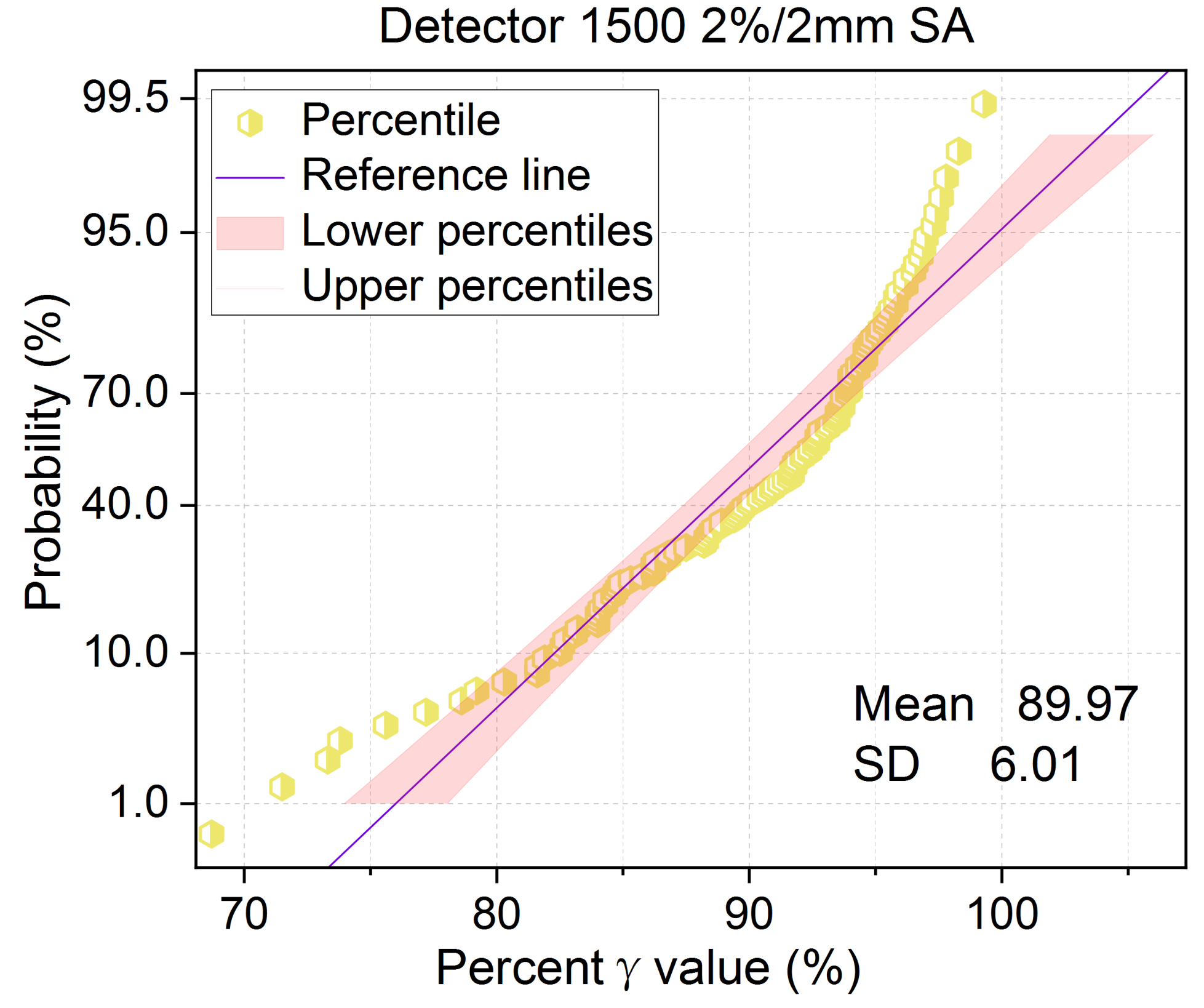


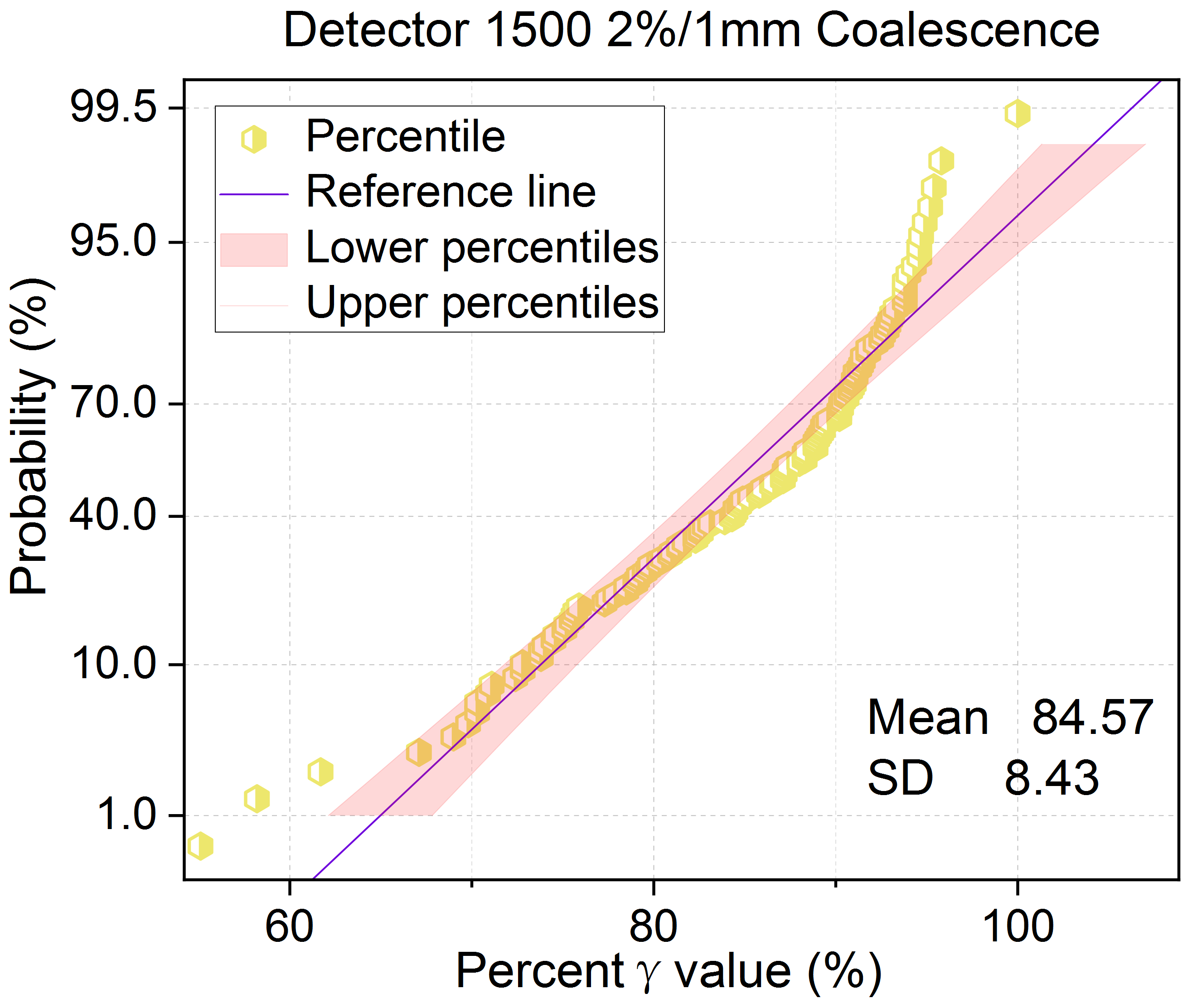

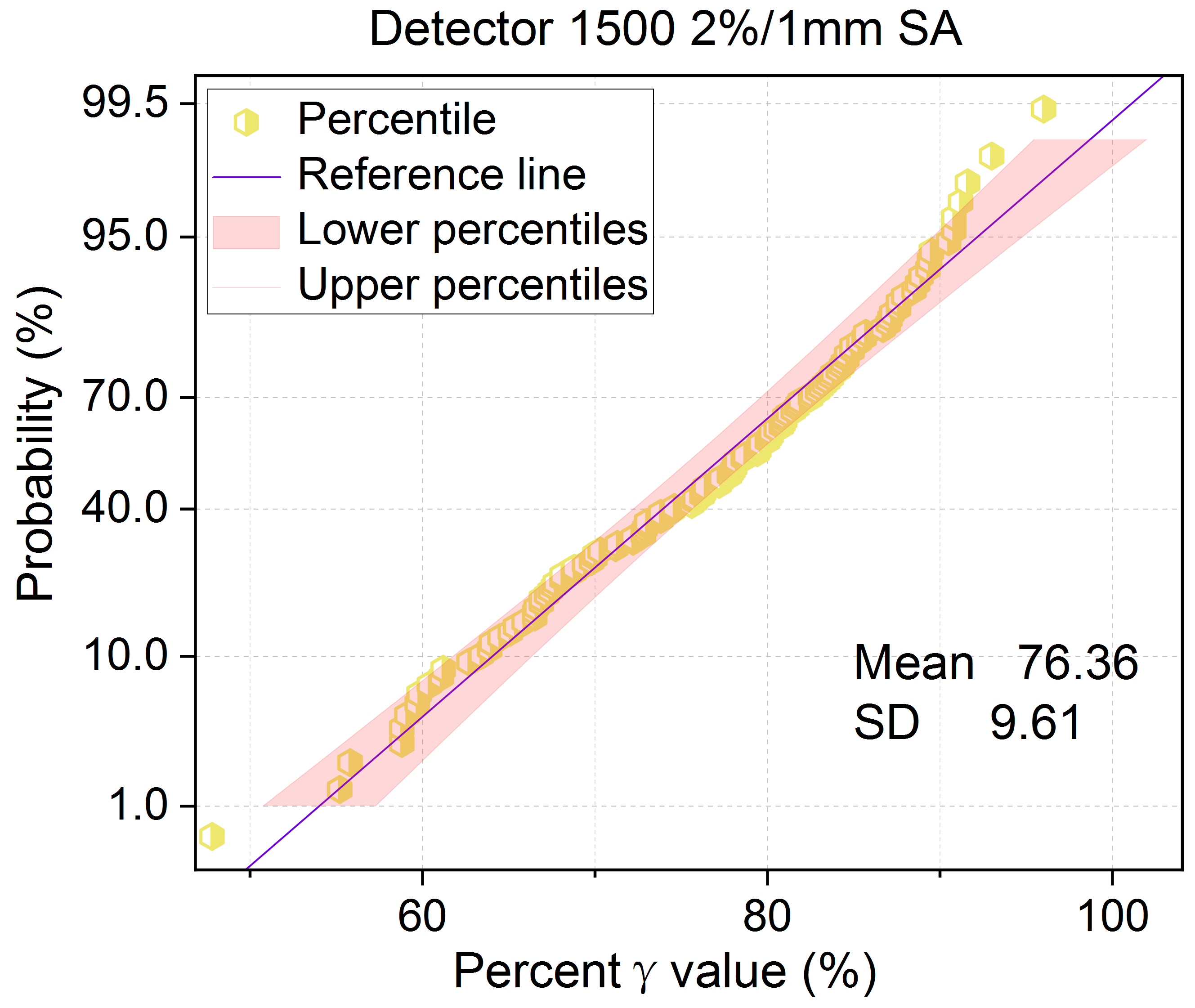

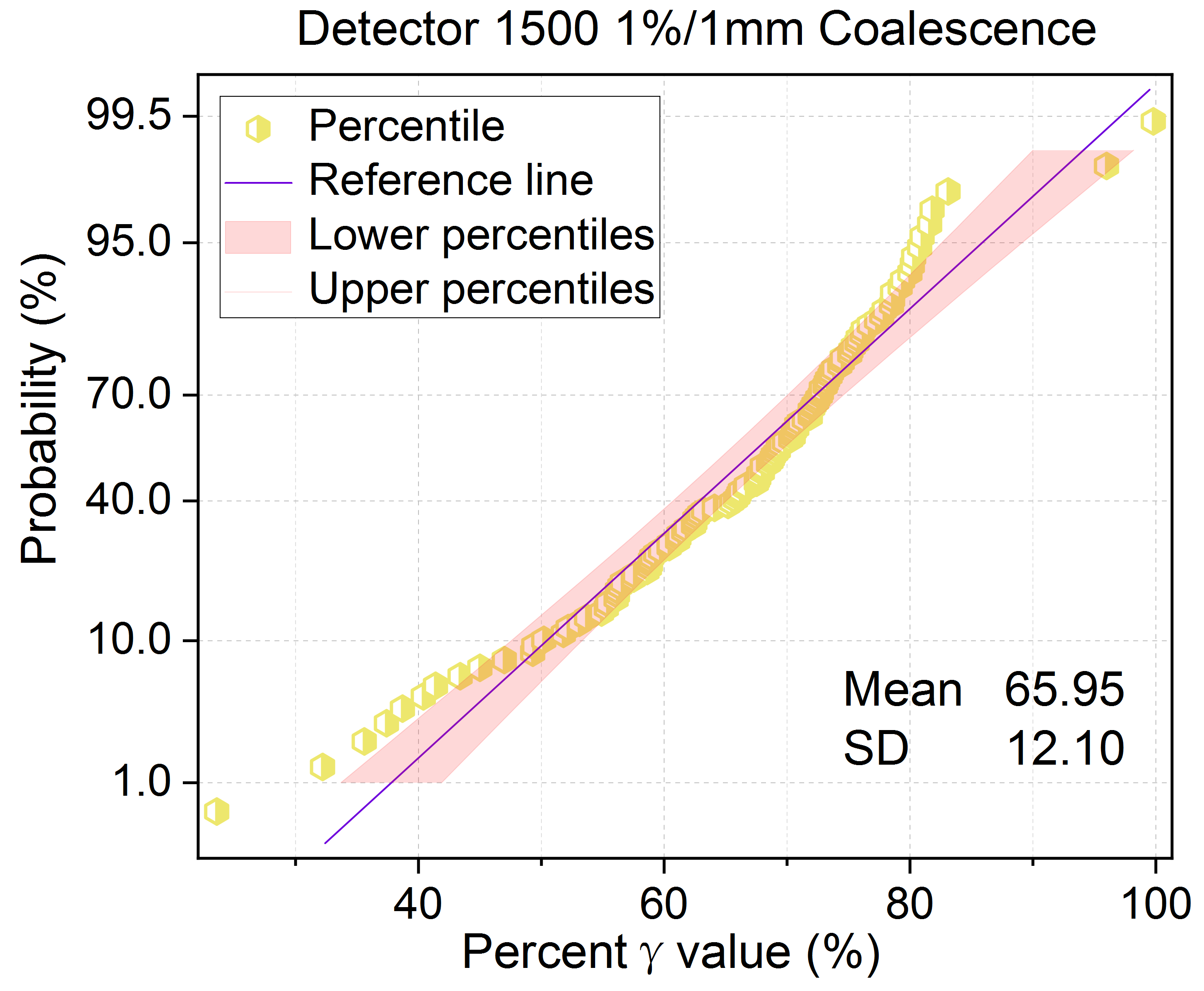

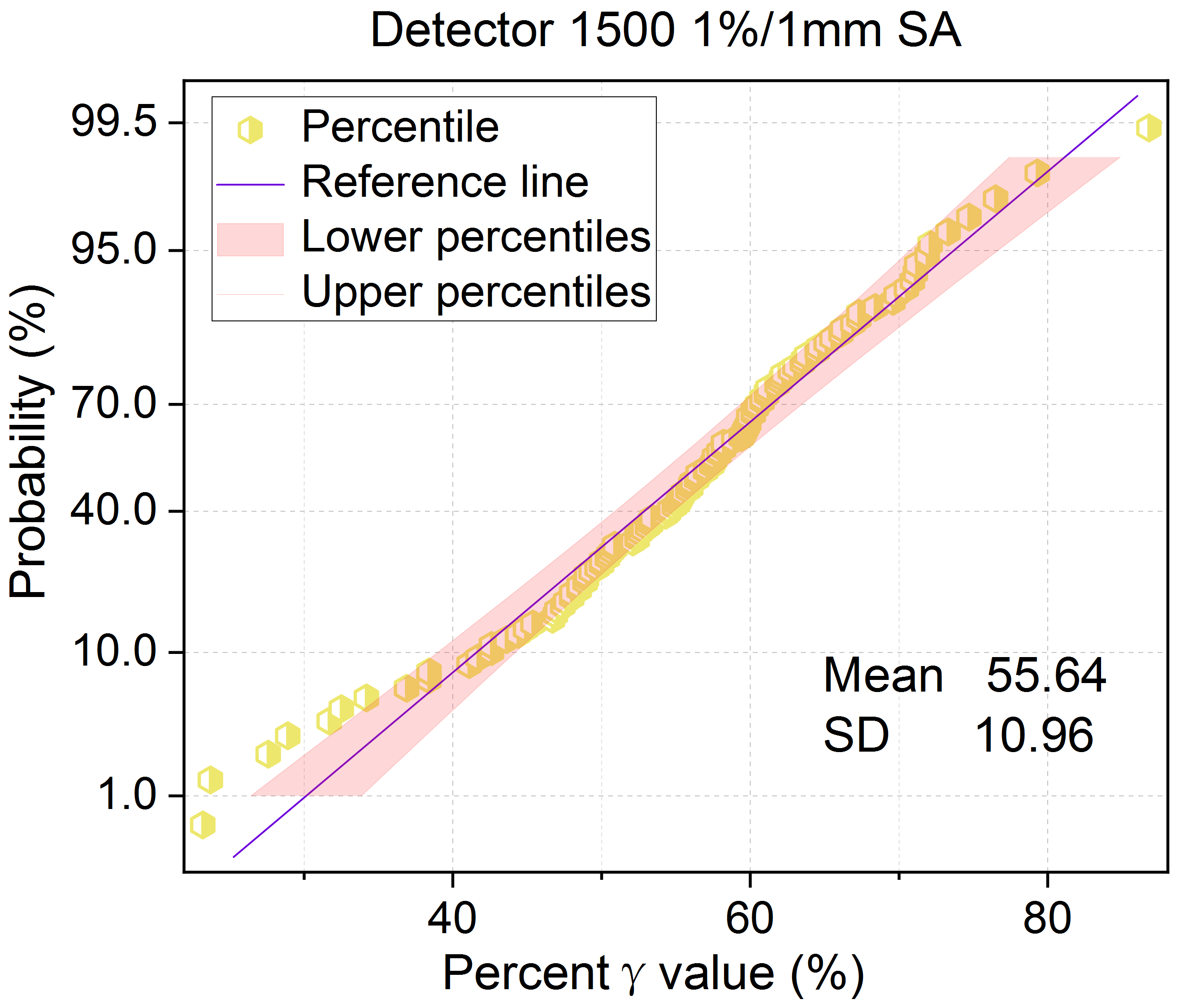


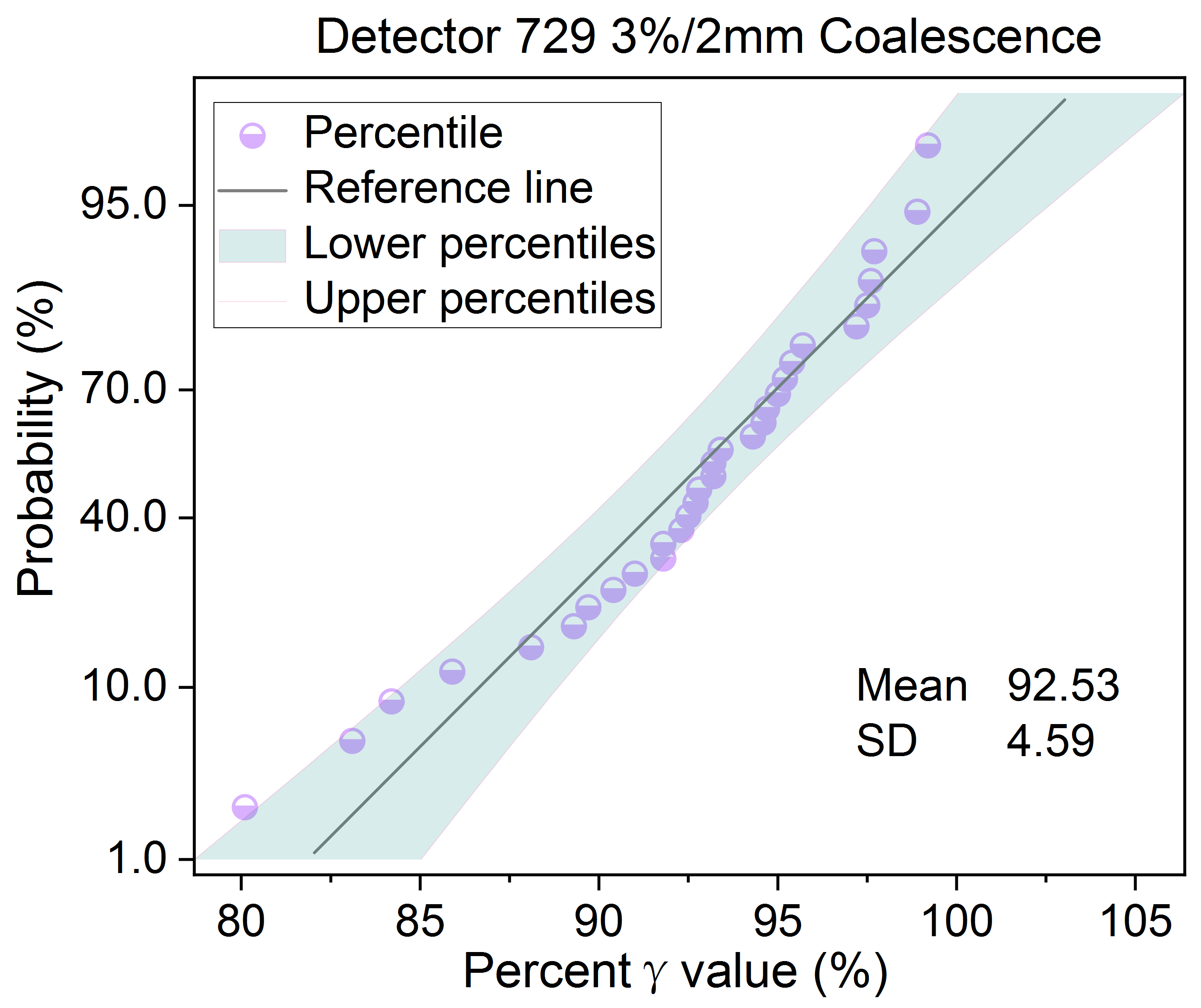

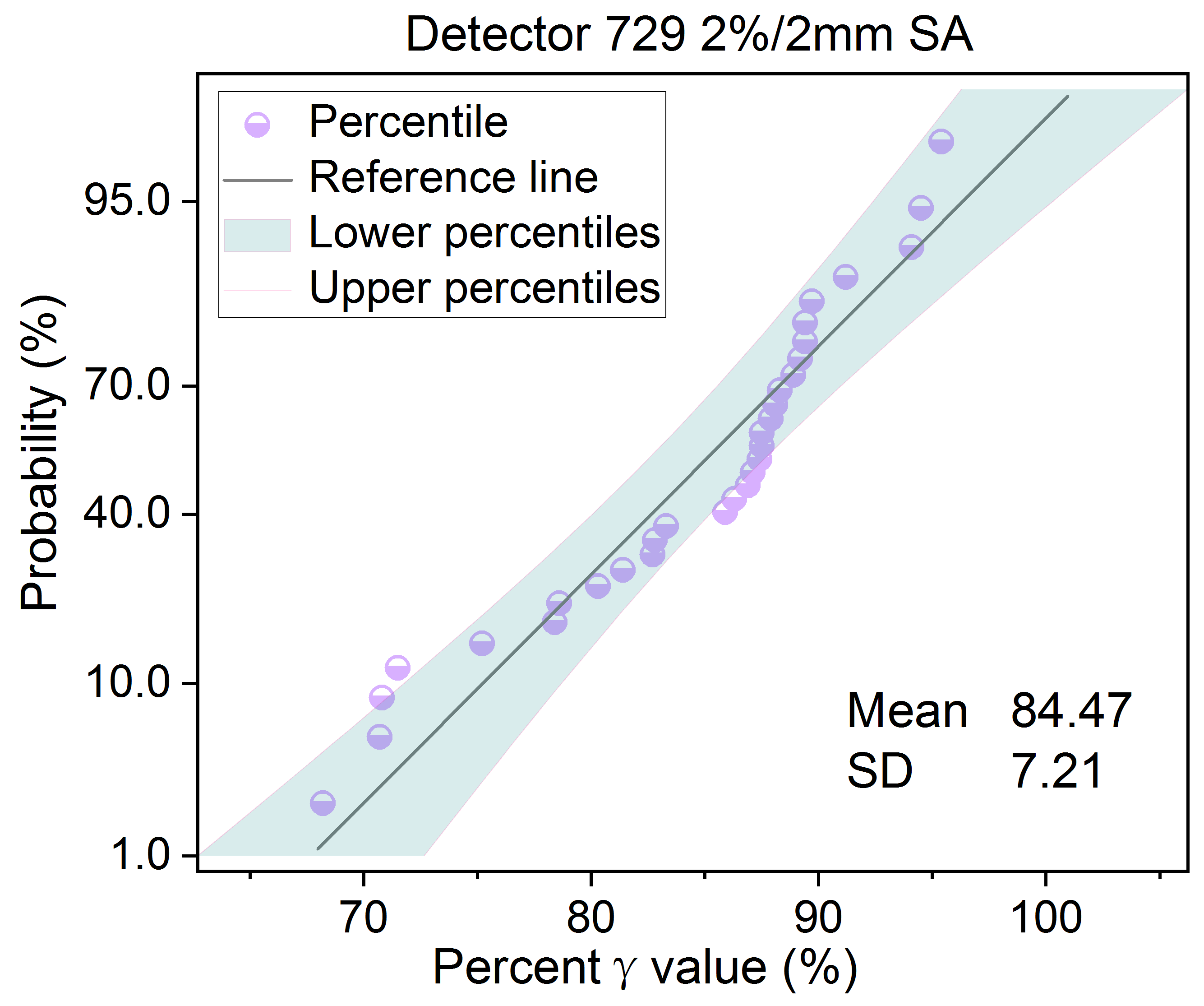

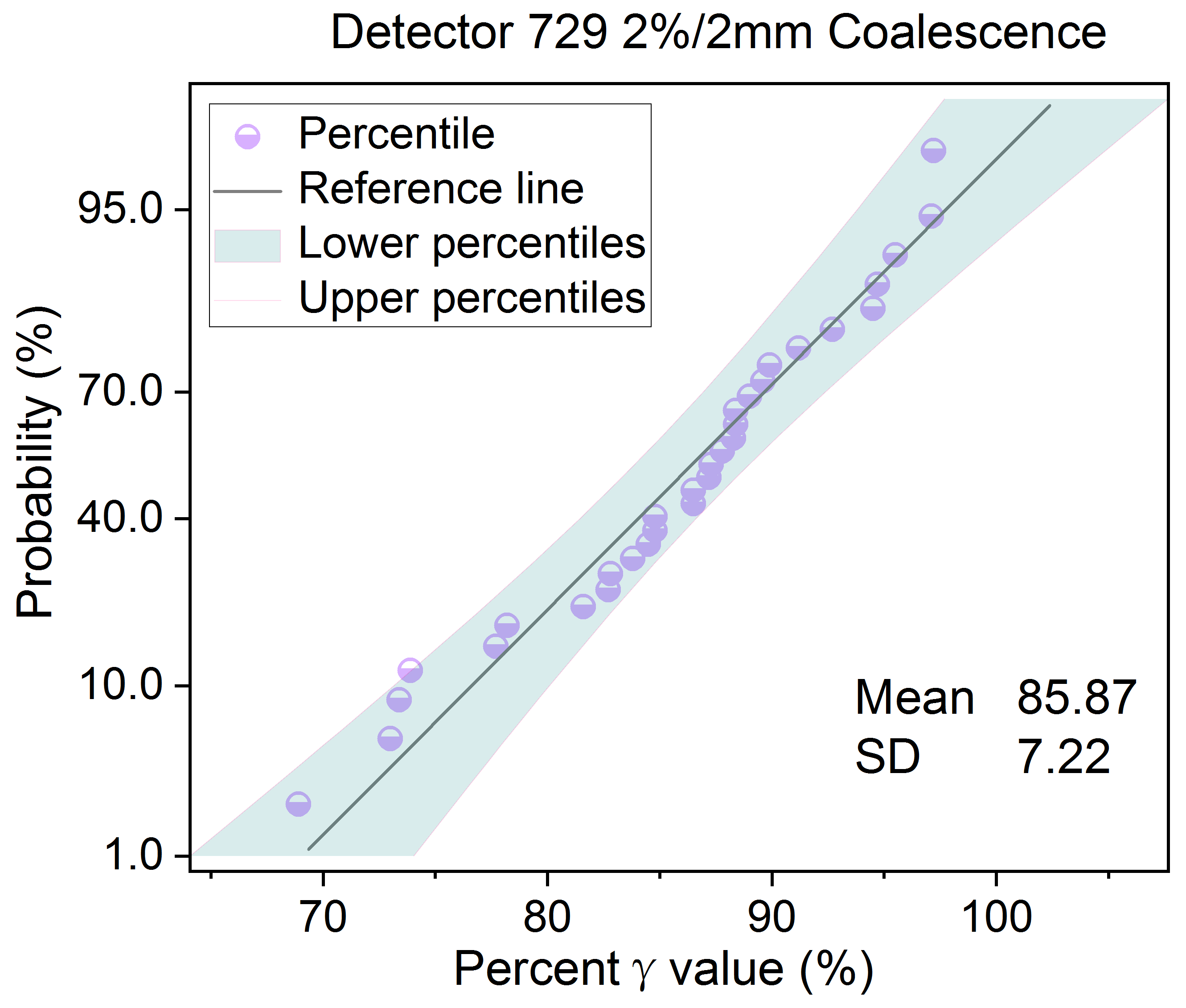

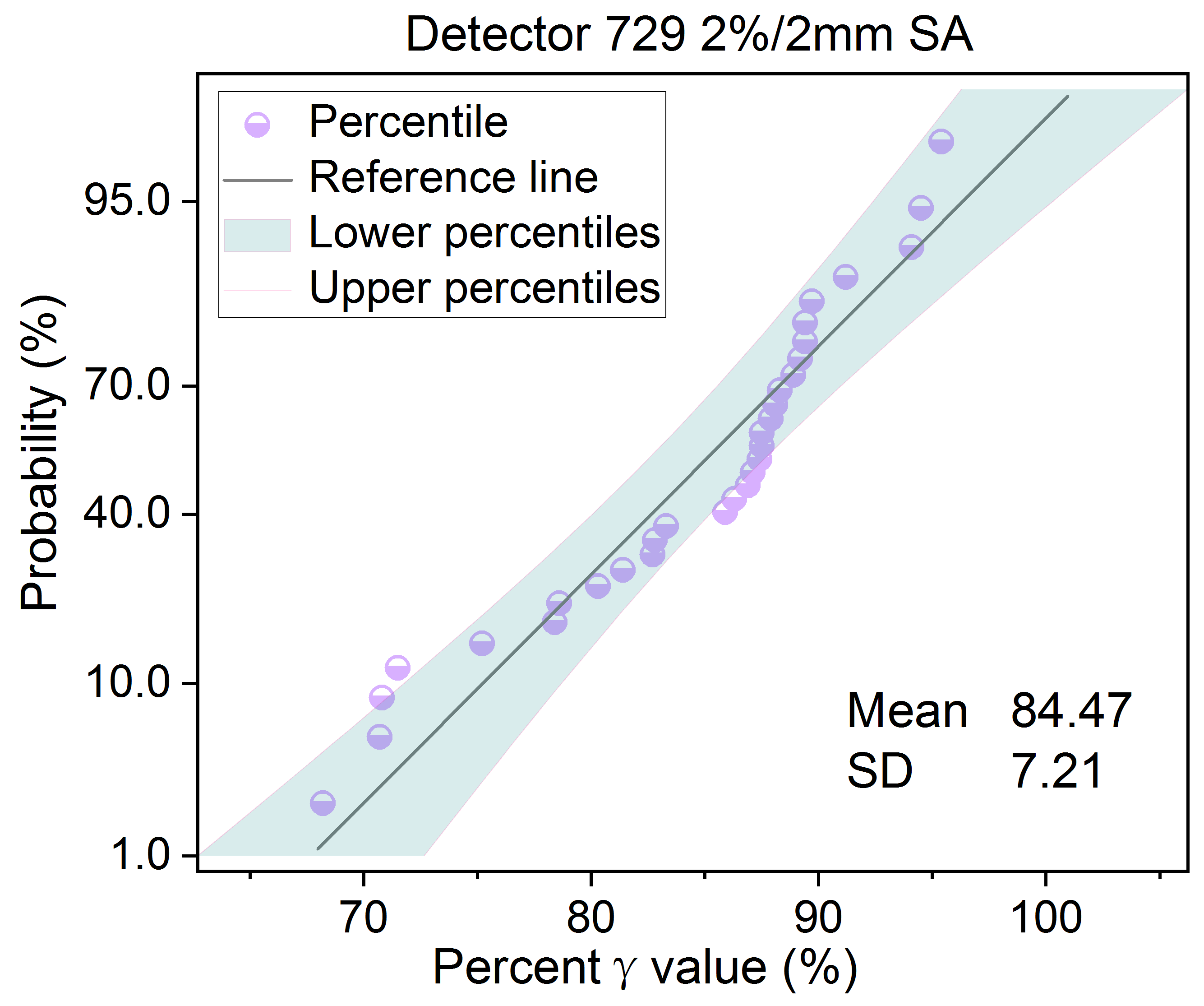


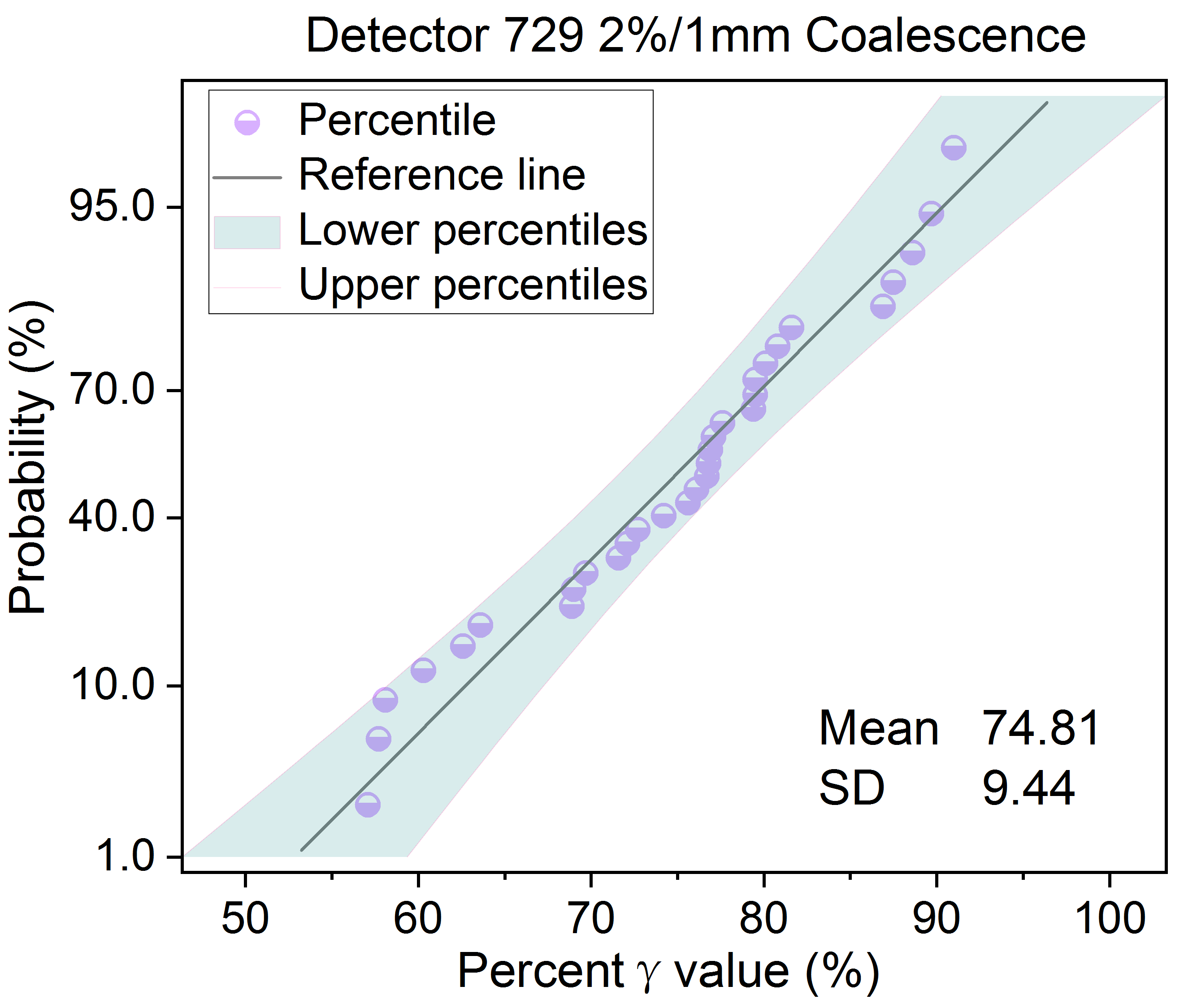

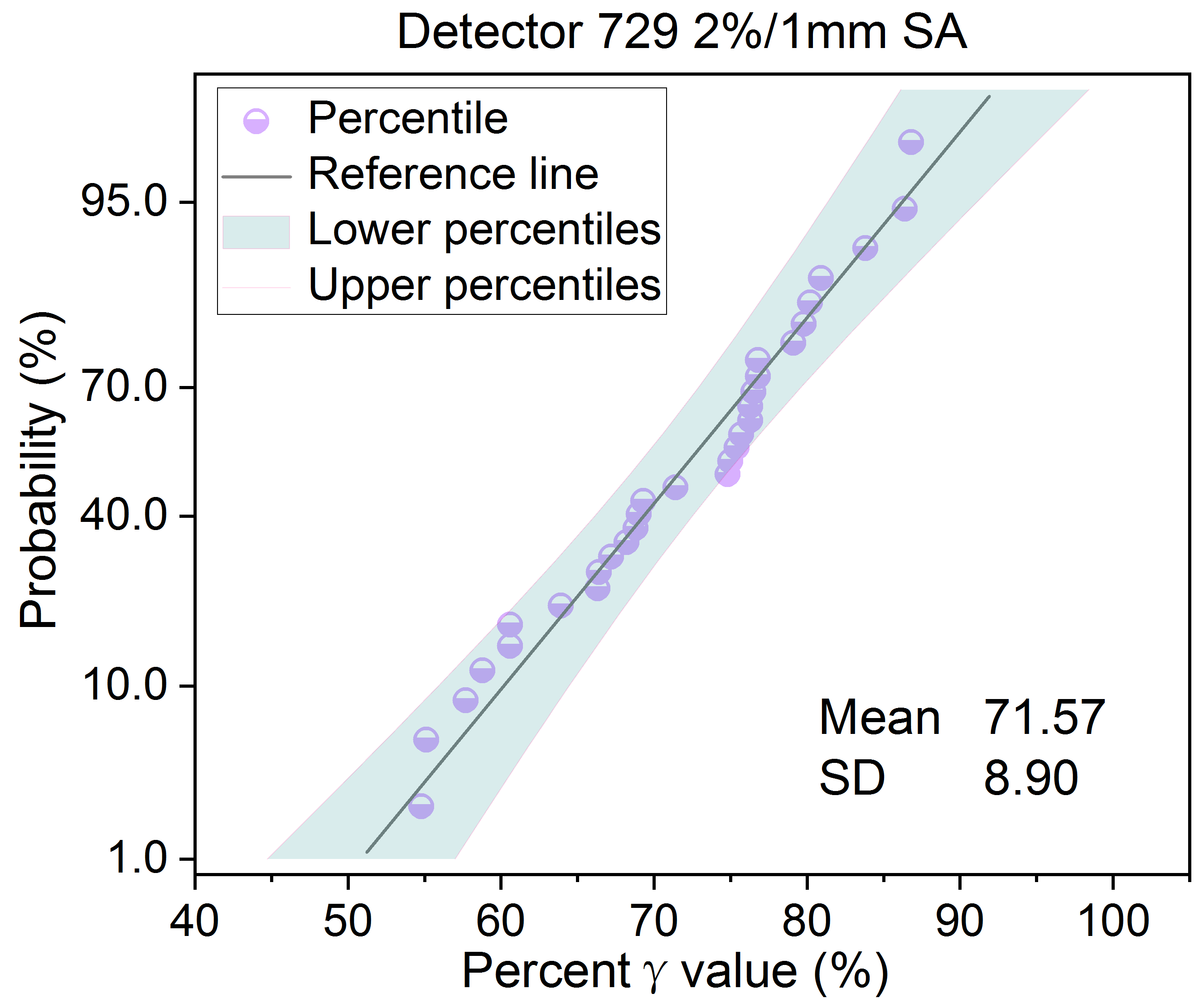

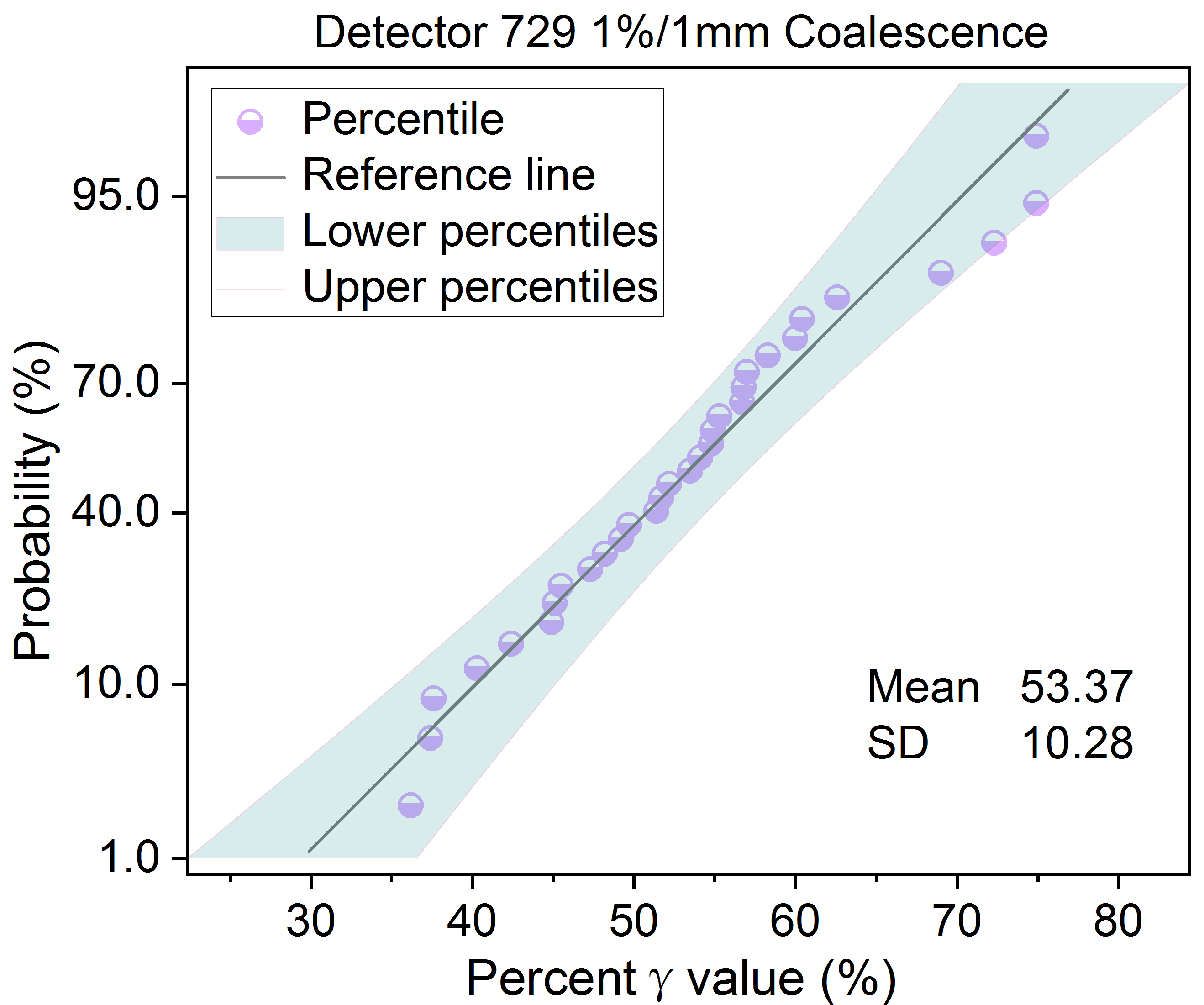

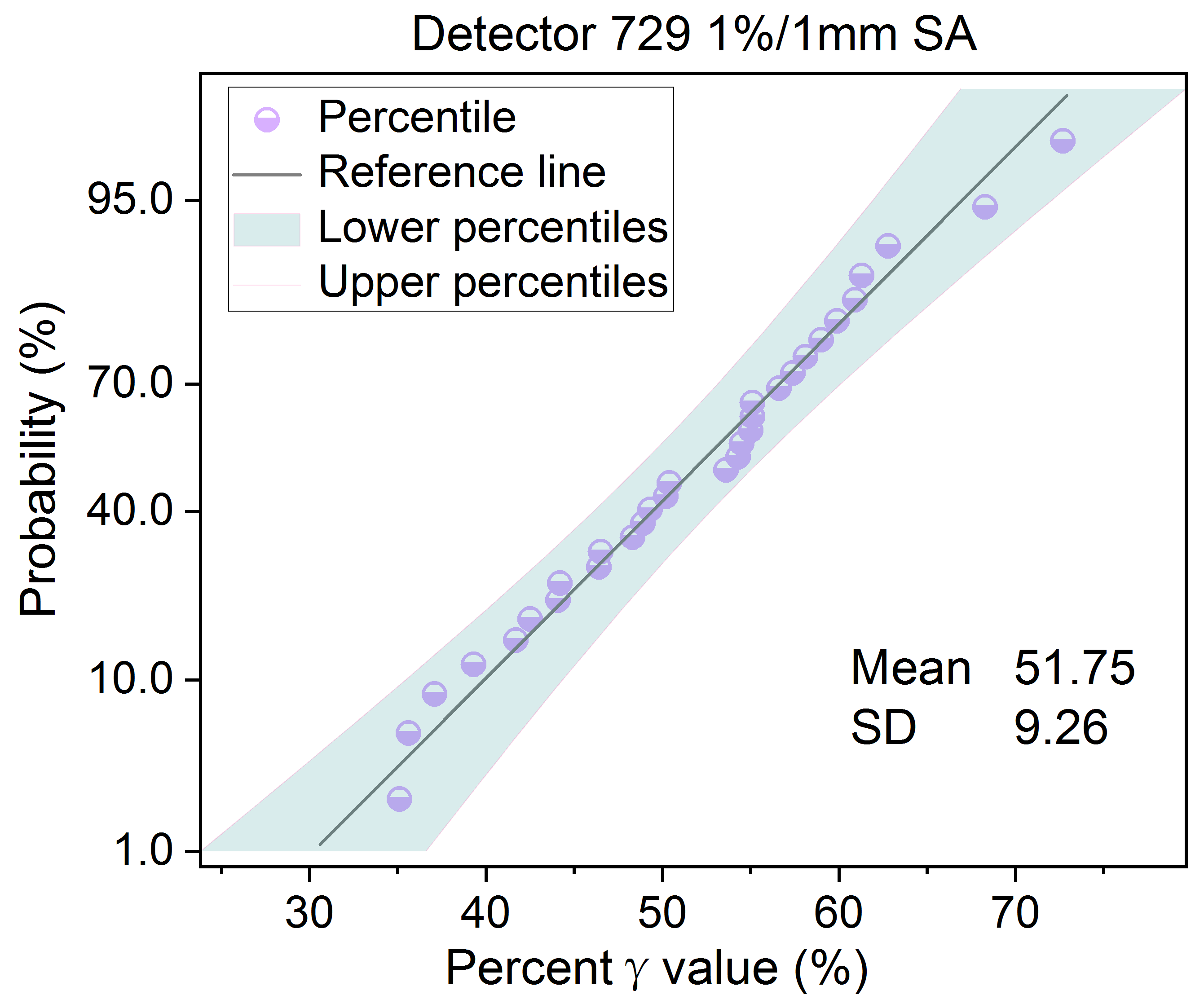


Normal probability plots of γ values in coalescence and SA cohorts for both detectors. Normal probability plots were generated to verify the assumption of approximately normally distributed data. In case of a normal distribution, the data plotted should form a straight line that corresponds to a theoretical normal distribution. Additionally, the Kolmogorov-Smirnov significance hypothesis test (K-S test) and Shapiro-Wilk test were used for each data set at different acceptance criteria to test whether one data set conformed to normally distribution (significant level=0.05). These probability plots verified the assumption of approximately normally distributed data for detector 1500 coalescence cohorts at 1%/1 mm criteria, SA cohorts at 2%/1mm and 1%/1mm criteria, and all samples of detector 729 conformed to a normal distribution (*P*>0.05, two-tailed). Others revealed violations of Normality assumptions (*P*<0.05, two-tailed). The straight line corresponds to a theoretical normal probability distribution for each data set.

Figure S4. Dose profile comparison of transverse view between measured and calculated dose distributions for a pelvic 6 MV VMAT plan.


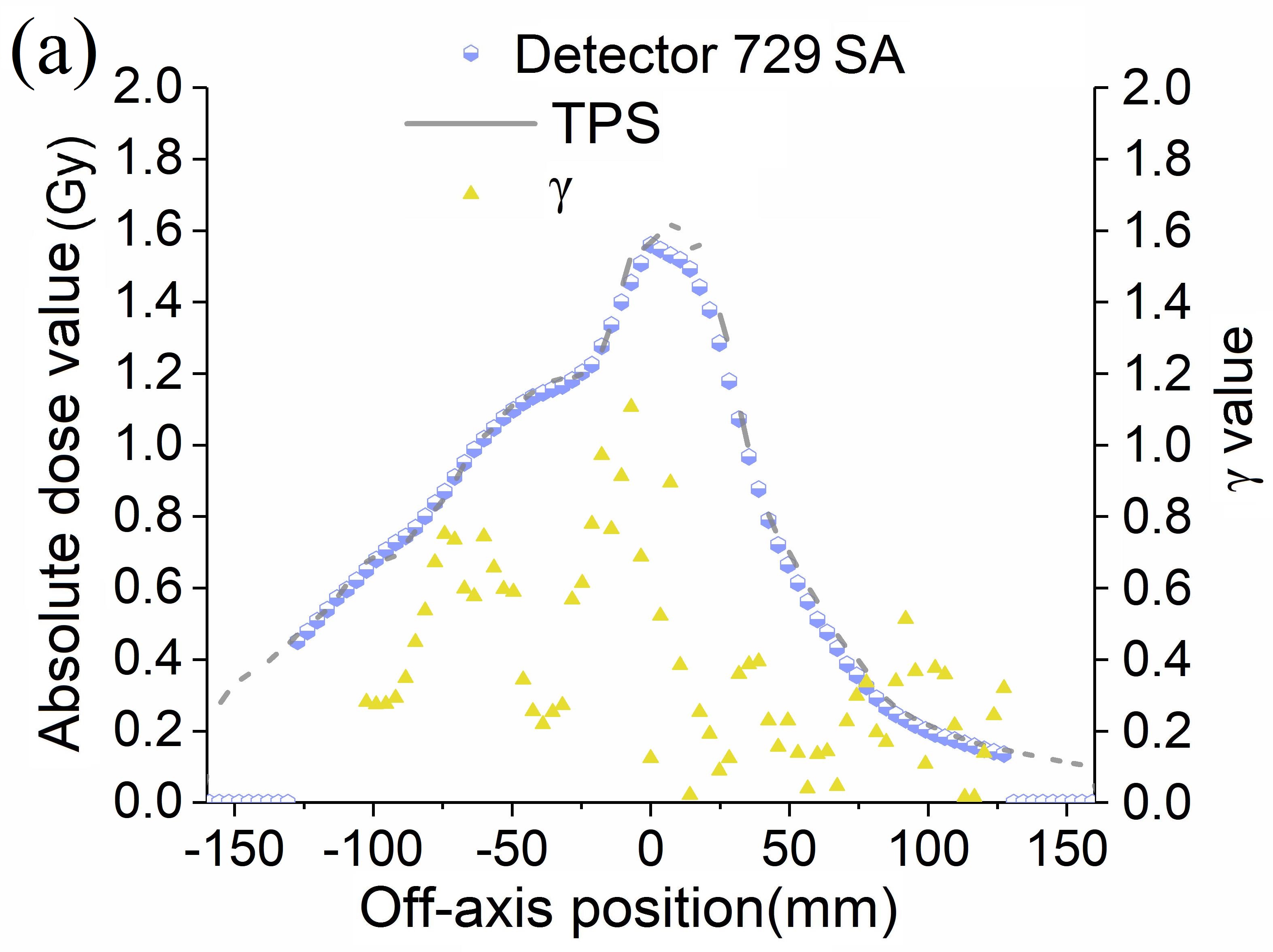

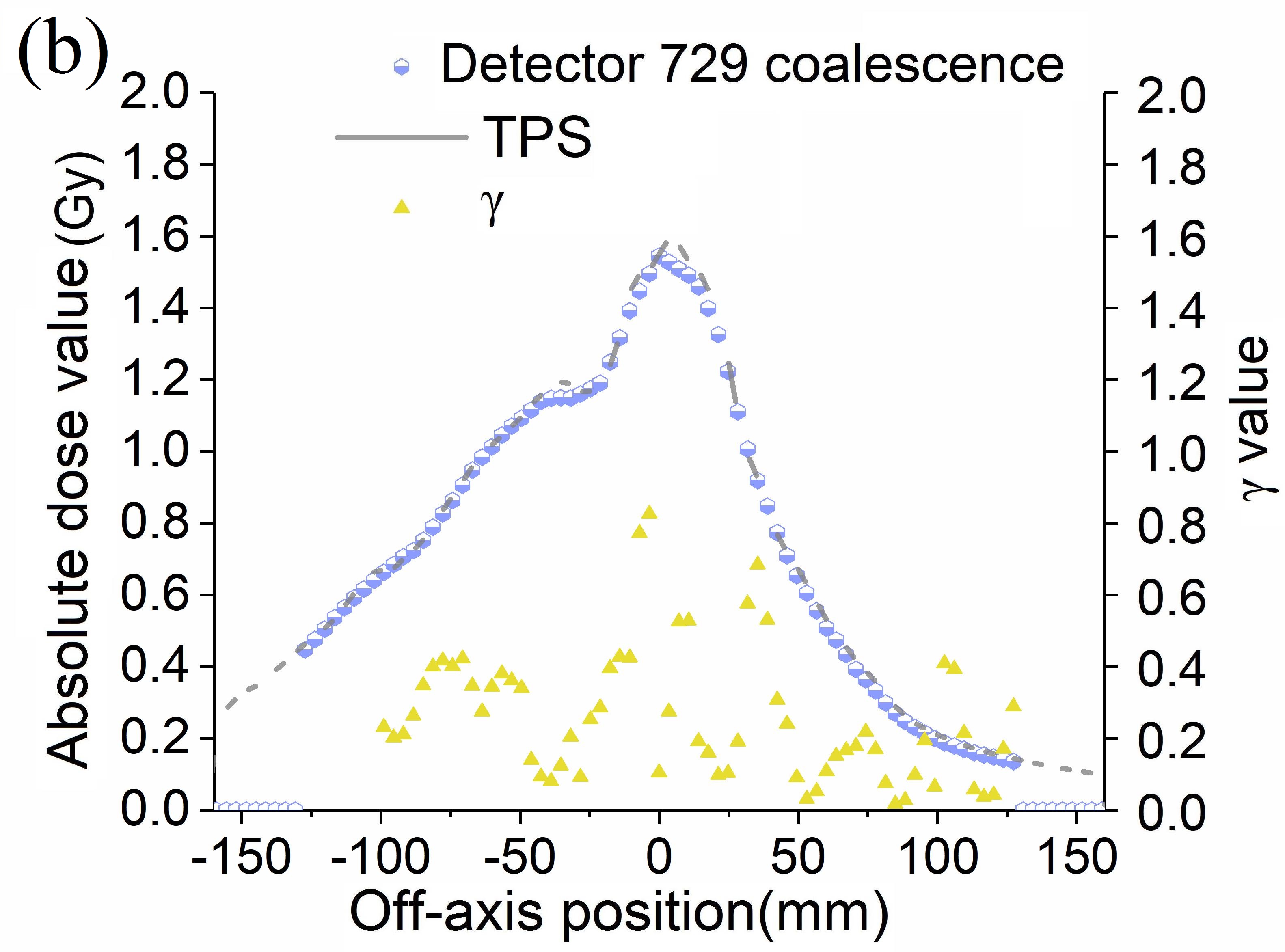

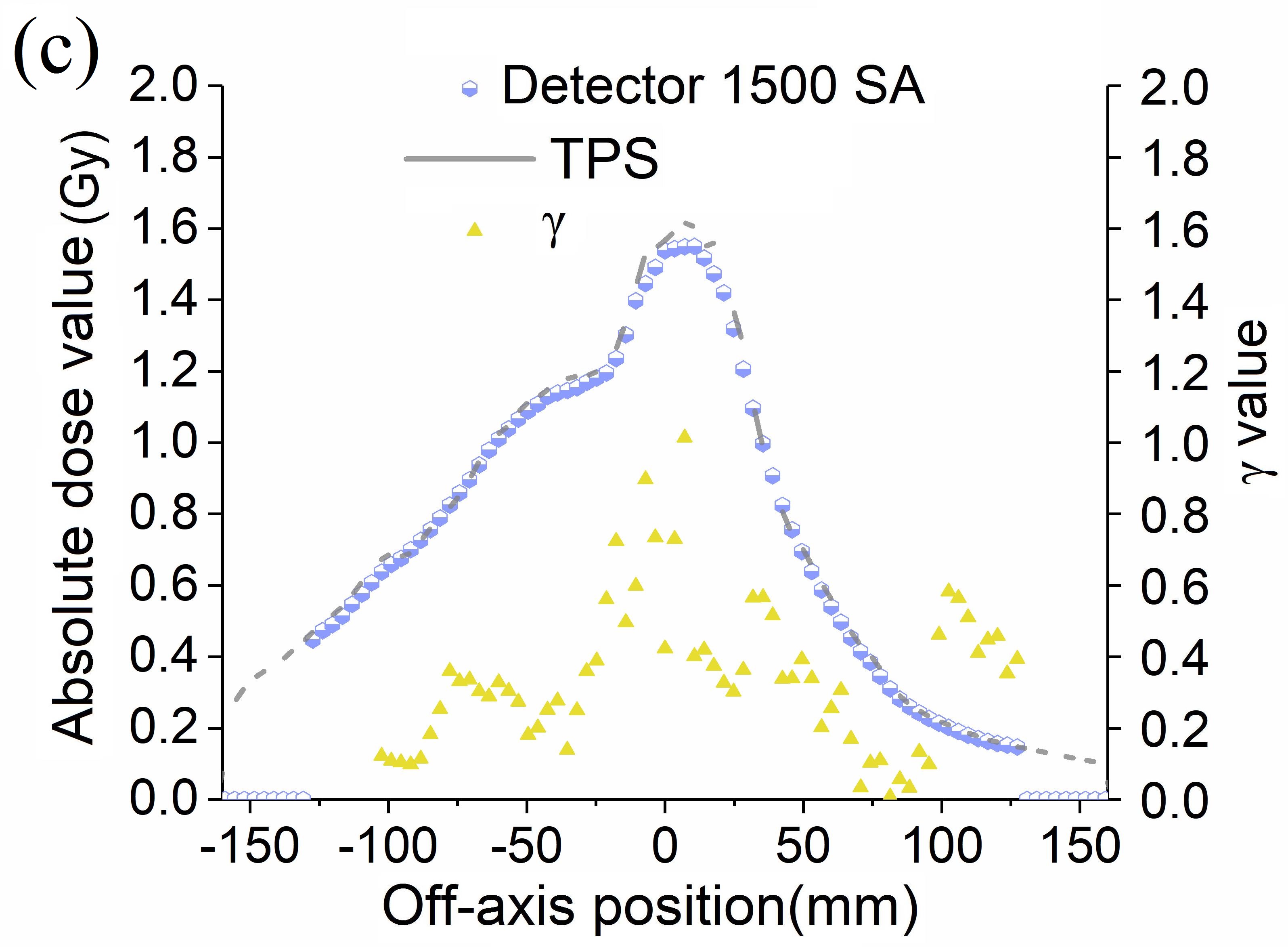

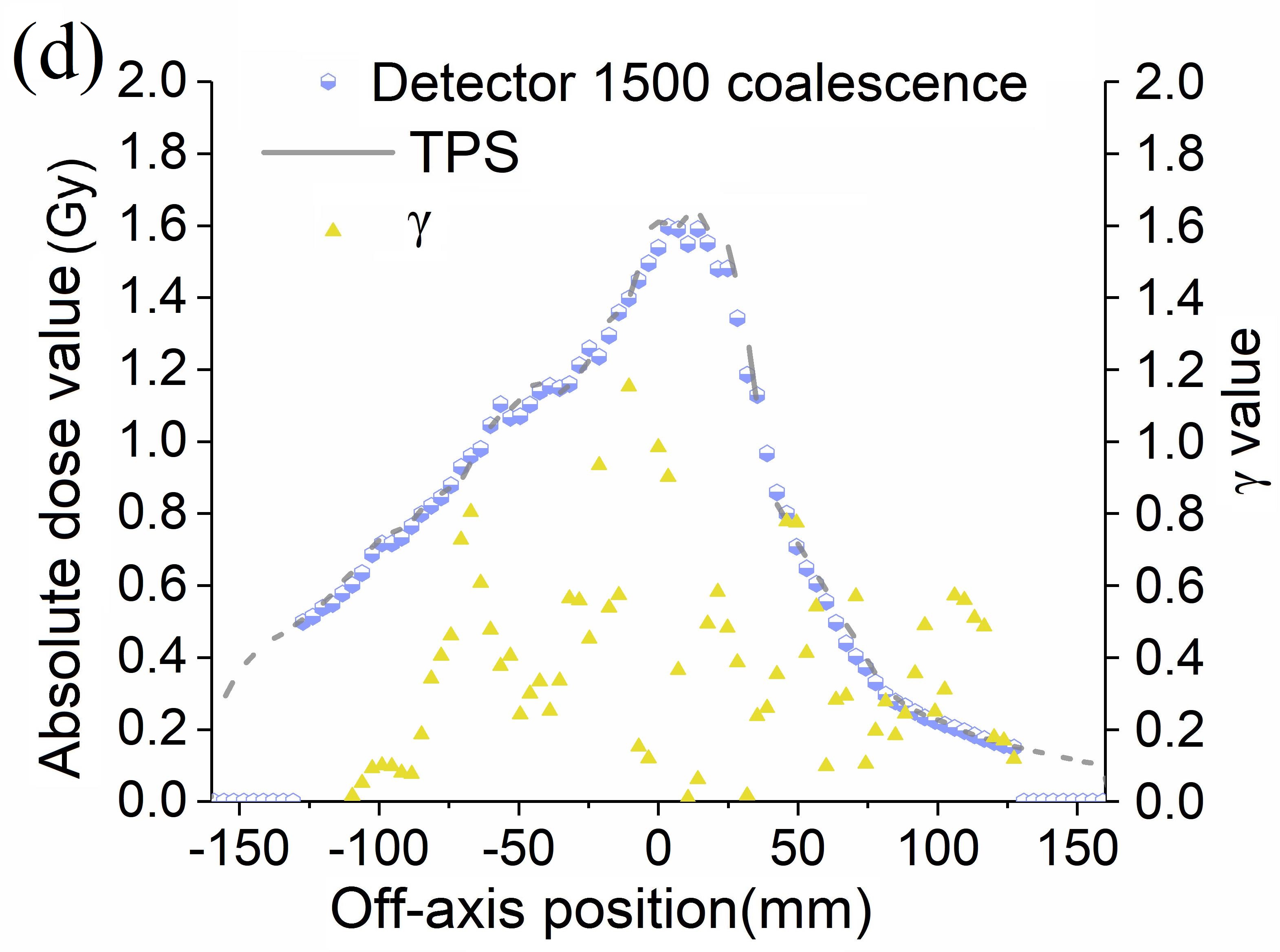


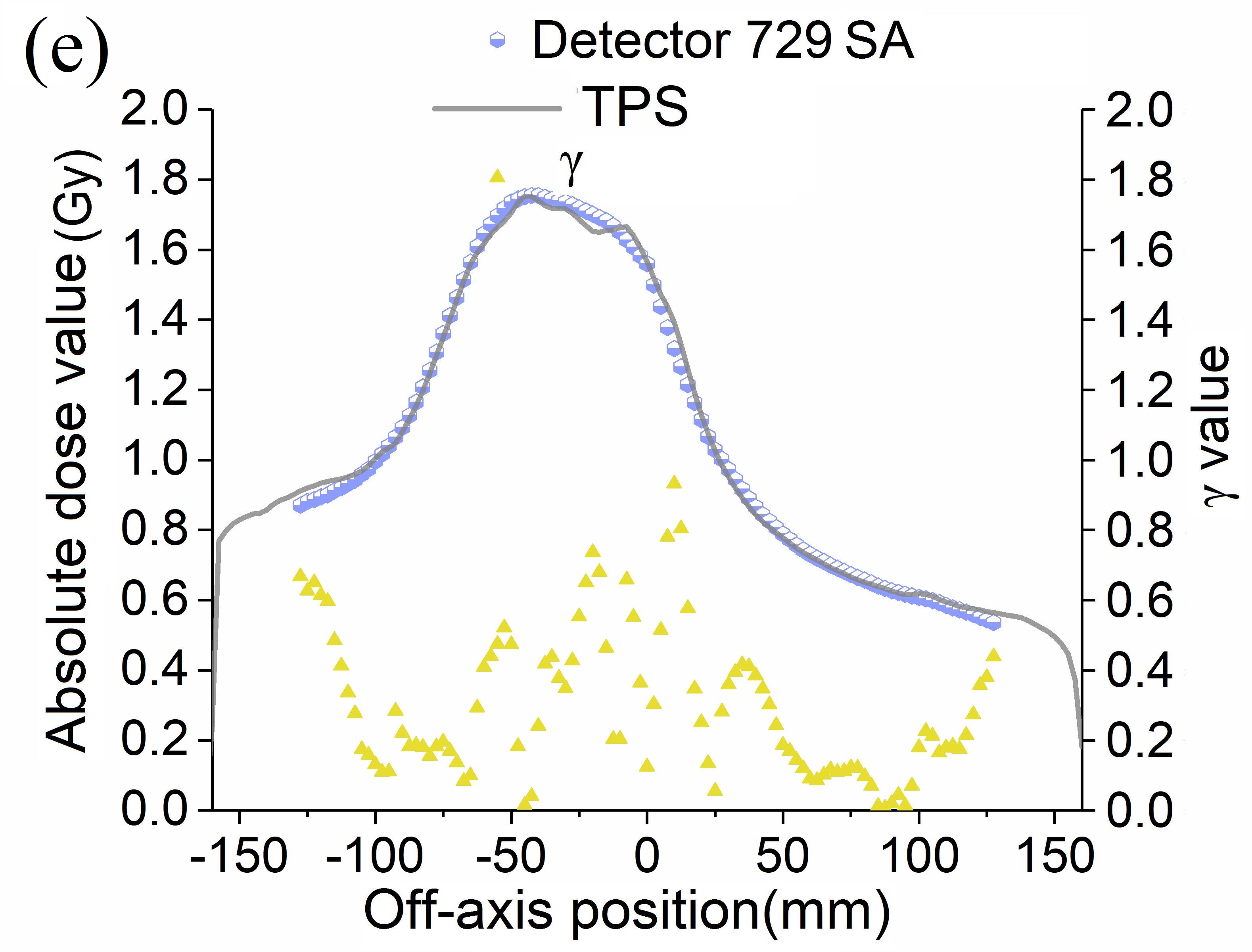

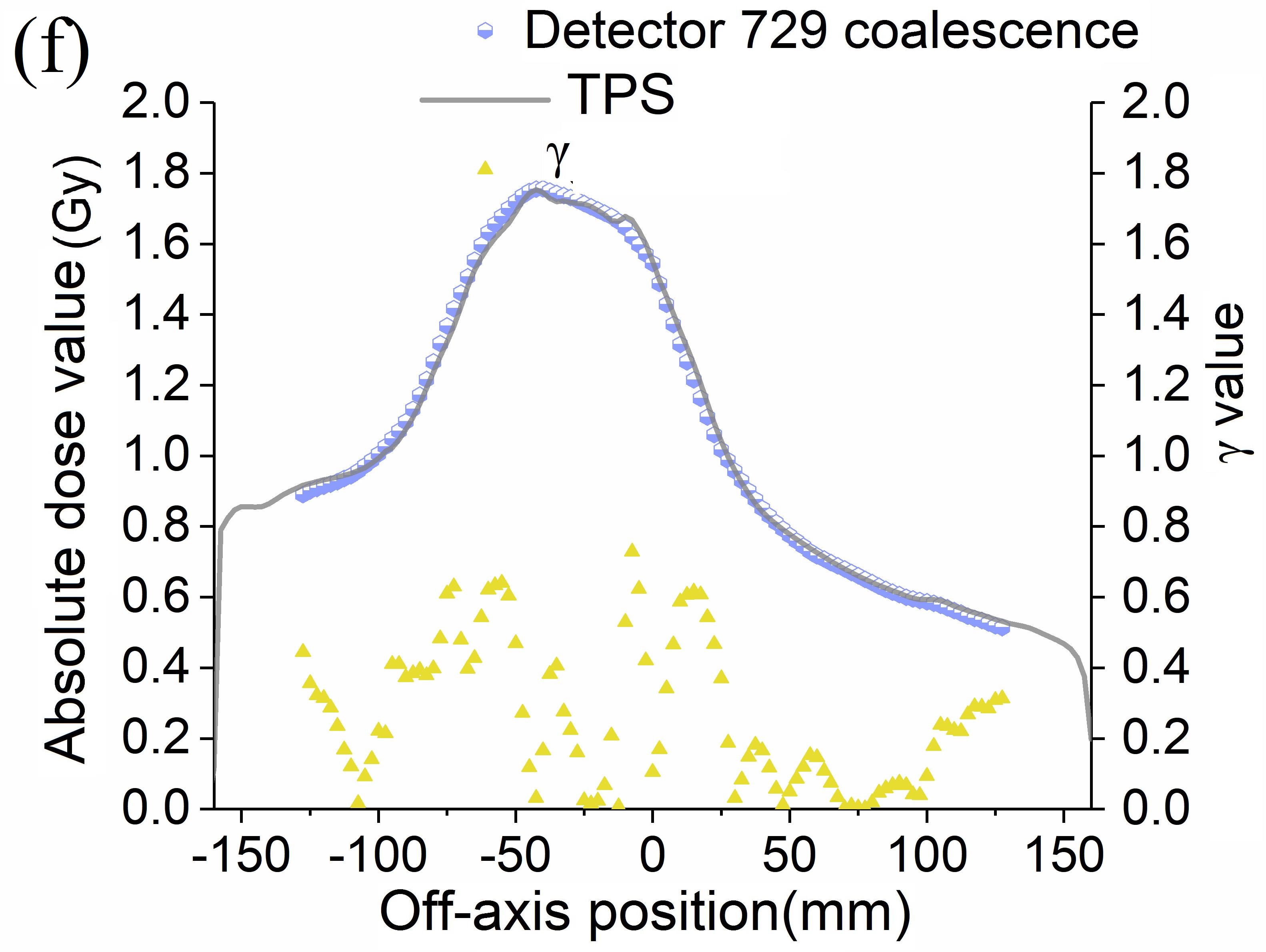

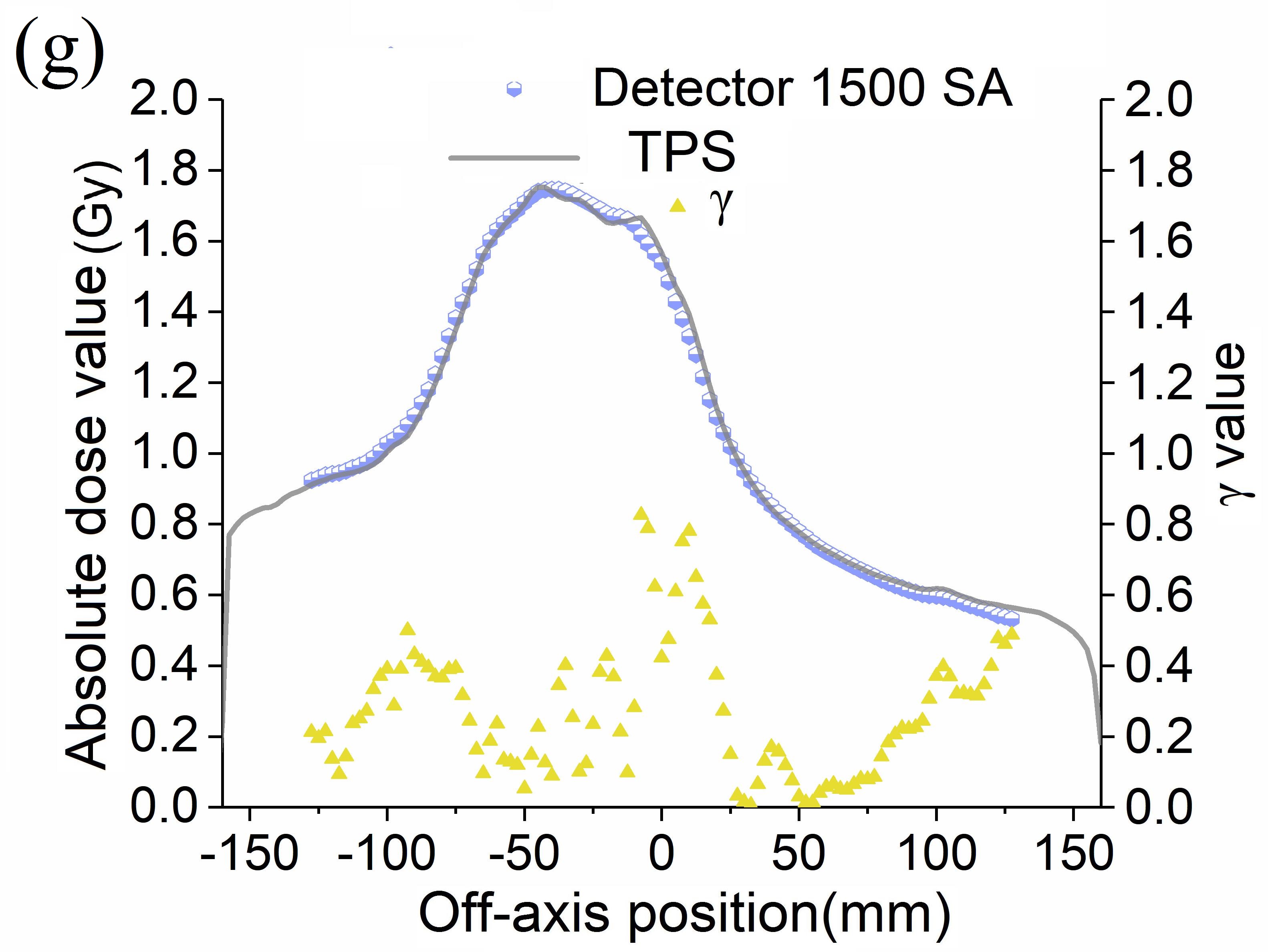

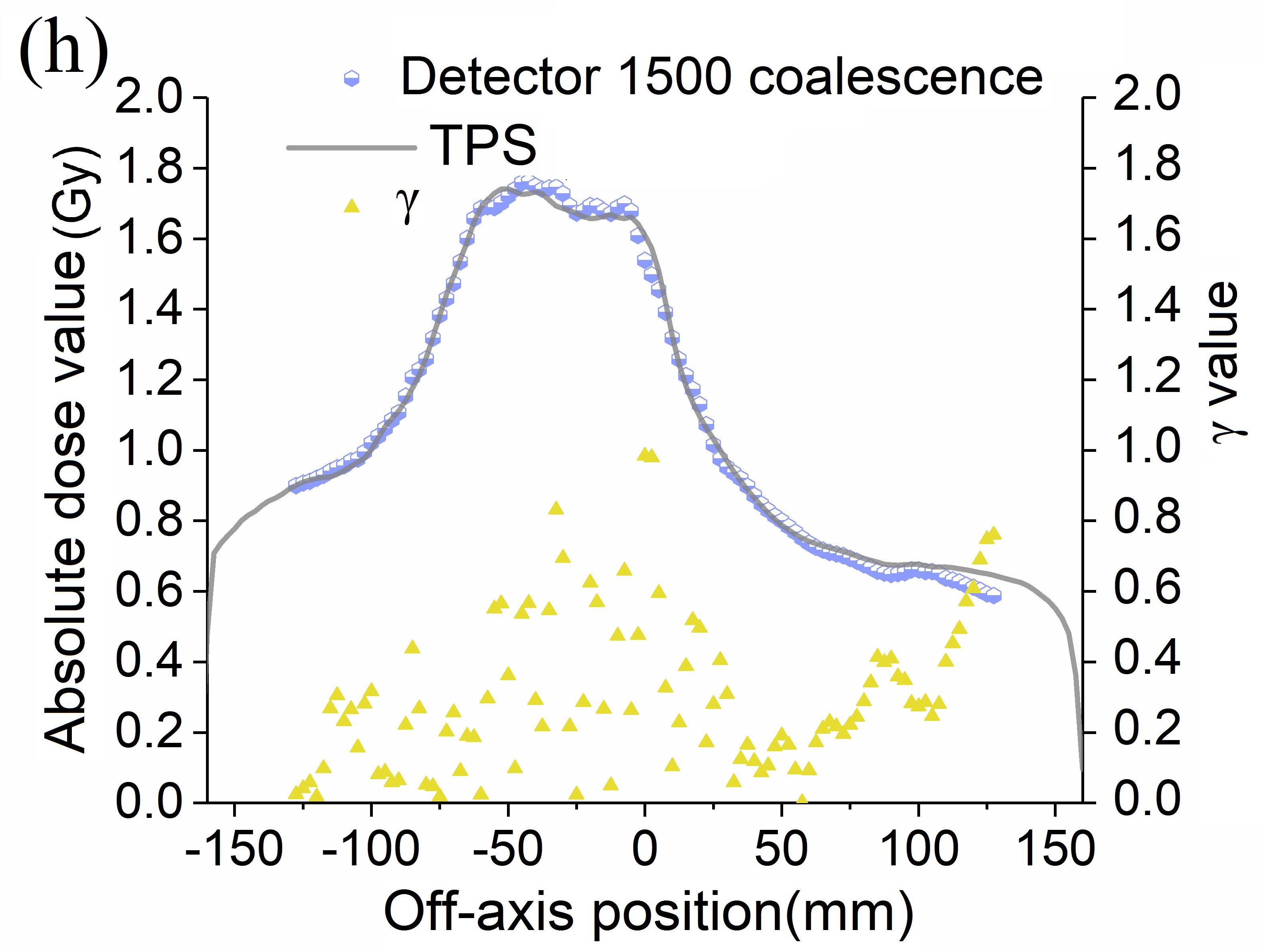


Dose profile comparison of transverse view between measured and calculated dose distribution in a pelvic 6 MV VMAT plan. (a-d) Dosimetric profile representations of the SA and coalescence cohorts in detectors along negative slope diagonal (from the upper right to the lower left of the detector). (e-h) Dosimetric profile representations of the SA and coalescence cohorts in detectors along the longitudinal forward and backward directions.

Table S1 Statistical results of γ mean difference for detector 729 at different acceptance criteria

| Tolerance criteria | Coalescence (%) | SA (%) | *Z/t* value | *P* value |
| --- | --- | --- | --- | --- |
| 3%/2mm | 92.70±4.23 | 91.52±4.30 | 2.225 | 0.034 |
| 2%/2mm | 85.49±7.24 | 84.48±6.55 | 1.709 | 0.098 |
| 2%/1mm | 75.07±8.88 | 71.58±7.90 | 3.149 | 0.004 |
| 1%1mm | 52.75±10.10 | 51.86±8.53 | 0.927 | 0.362 |

Table S2 Statistical results of γ mean difference for detector 1500 at different acceptance criteria

| Tolerance criteria | Coalescence (%) | SA (%) | *Z/t* value | *P* value |
| --- | --- | --- | --- | --- |
| 3%/2mm | 97.87±2.31 | 95.43±3.27 | -8.08 | 0.000 |
| 2%/2mm | 94.78±4.84 | 89.90±6.19 | -8.238 | 0.000 |
| 2%/1mm | 84.47±8.67 | 76.24±9.83 | -8.205 | 0.000 |
| 1%1mm | 65.85±12.23 | 55.43±11.32 | 11.890 | 0.000 |

The mean percent γ values and standard deviations in coalescence versus SA cohorts at different acceptance criteria. Statistical significance was determined by two-tailed paired t test and Wilcoxon signed rank test. *P*<0.05 indicates a statistically significant difference, and vice versa. In the paired t-test, the coalescence cohorts were the control groups and the SA cohorts were the study groups, and vice versa in Wilcoxon signed rank test. When the averages in study groups were smaller than those in the control groups, the t or T value was larger than 0. The larger the absolute value of t or T, the larger the difference of paired sample mean.

Table S3 Statistical results of γ values

| Cohorts | Tolerance criteria | Mean (%) | SD | Median (%) | Range (%) | Variance |
| --- | --- | --- | --- | --- | --- | --- |
| Detector 729 Coalescence | 3%/2mm | 92.70 | 4.23 | 93.15 | 80.1 ~ 99.2 | 17.86 |
|  | 2%/2mm | 85.49 | 7.24 | 86.85 | 68.3 ~ 97.2 | 52.45 |
|  | 2%/1mm | 75.07 | 8.88 | 76.75 | 57.1 ~ 91.0 | 78.86 |
|  | 1%1mm | 52.75 | 10.10 | 52.60 | 36.2 ~ 74.9 | 102.01 |
| Detector 729 SA | 3%/2mm | 91.52 | 4.30 | 92.90 | 81.2 ~ 97.7 | 18.45 |
|  | 2%/2mm | 84.48 | 6.55 | 87.00 | 70.7 ~ 95.4 | 42.88 |
|  | 2%/1mm | 71.58 | 7.90 | 73.10 | 55.1 ~ 86.8 | 62.37 |
|  | 1%1mm | 51.86 | 8.53 | 53.95 | 35.1 ~ 72.7 | 72.79 |
| Detector 1500 Coalescence | 3%/2mm | 97.87 | 2.31 | 98.70 | 88.9 ~ 100 | 5.33 |
|  | 2%/2mm | 94.78 | 4.84 | 96.70 | 74.6 ~ 100 | 23.38 |
|  | 2%/1mm | 84.47 | 8.67 | 87.10 | 55.1 ~100 | 75.16 |
|  | 1%1mm | 65.85 | 12.23 | 68.00 | 23.6 ~ 99.8 | 149.64 |
| Detector 1500 SA | 3%/2mm | 95.43 | 3.27 | 96.30 | 82.7 ~ 99.8 | 10.67 |
|  | 2%/2mm | 89.90 | 6.19 | 91.70 | 68.7 ~ 99.3 | 38.27 |
|  | 2%/1mm | 76.24 | 9.83 | 77.90 | 47.8 ~ 96.0 | 96.62 |
|  | 1%1mm | 55.43 | 11.32 | 56.50 | 23.2 ~ 86.8 | 128.05 |
